# Efficacy and completion rates of rifapentine and isoniazid (3HP) compared to other treatment regimens for latent tuberculosis infection: a systematic review with network meta-analyses

**DOI:** 10.1186/s12879-017-2377-x

**Published:** 2017-04-11

**Authors:** Christopher Pease, Brian Hutton, Fatemeh Yazdi, Dianna Wolfe, Candyce Hamel, Pauline Quach, Becky Skidmore, David Moher, Gonzalo G. Alvarez

**Affiliations:** 1 Department of Medicine, The Ottawa Hospital, Ottawa, Canada; 2 Ottawa Hospital Research Institute, Ottawa, ON K1H 8L6 Canada; 3 Ottawa University School of Epidemiology, Public Health and Preventive Medicine, Ottawa, Canada; 4 Ottawa University Faculty of Medicine, Ottawa, Canada

**Keywords:** Systematic review, Network meta-analysis, Latent tuberculosis infection

## Abstract

**Background:**

We conducted a systematic review and network meta-analysis (NMA) to examine the efficacy and completion rates of treatments for latent tuberculosis infection (LTBI). While a previous review found newer, short-duration regimens to be effective, several included studies did not confirm LTBI, and analyses did not account for variable follow-up or assess completion.

**Methods:**

We searched MEDLINE, Embase, CENTRAL, PubMed, and additional sources to identify RCTs in patients with confirmed LTBI that involved a regimen of interest and reported on efficacy or completion. Regimens of interest included isoniazid (INH) with rifapentine once weekly for 12 weeks (INH/RPT-3), 6 and 9 months of daily INH (INH-6; INH-9), 3–4 months daily INH plus rifampicin (INH/RFMP 3–4), and 4 months daily rifampicin alone (RFMP-4). NMAs were performed to compare regimens for both endpoints.

**Results:**

Sixteen RCTs (*n* = 44,149) and 14 RCTs (*n* = 44,128) were included in analyses of efficacy and completion. Studies were published between 1968 and 2015, and there was diversity in patient age and comorbidities. All regimens of interest except INH-9 showed significant benefits in preventing active TB compared to placebo. Comparisons between active regimens did not reveal significant differences. While definitions of regimen completion varied across studies, regimens of 3–4 months were associated with a greater likelihood of adequate completion.

**Conclusions:**

Most of the active regimens showed an ability to reduce the risk of active TB relative to no treatment, however important differences between active regimens were not found. Shorter rifamycin-based regimens may offer comparable benefits to longer INH regimens. Regimens of 3–4 months duration are more likely to be completed than longer regimens.

**Electronic supplementary material:**

The online version of this article (doi:10.1186/s12879-017-2377-x) contains supplementary material, which is available to authorized users.

## Background

Tuberculosis (TB) is estimated to have infected one third of the world’s population [1] and resulted in the deaths of 1.5 million people in 2014 [2]. Not all individuals infected with TB develop active disease. [3] Many retain a population of viable *Mycobacterium tuberculosis* bacilli that is sequestered by the immune system, a state which is termed latent tuberculosis infection (LTBI). [4] Clinically, LTBI is defined by a persistent immune response to *M. tuberculosis* antigens without evidence of active disease [2]. Immune response can be assessed by both tuberculin skin testing (TST) and the interferon-gamma release assay (IGRA) [2]. Those with LTBI are neither infectious nor symptomatic, but are estimated to have a 5–15% lifetime risk of developing active TB from reactivation of their infection [3].

The goal of LTBI treatment is to reduce the risk of reactivation. Treatment with isoniazid (INH) is the traditional standard and regimens of 6 and 9 months of INH have been recommended [2]. However, the prolonged course of treatment required with INH has led to development of several alternative rifamycin-based regimens. The World Health Organization currently recommends the following regimens as options for LTBI treatment: 6 months of daily INH (INH-6), 9 month of daily INH (INH-9), INH and rifapentine once weekly for 12 weeks (INH/RPT-3), 3–4 months daily INH plus rifampin (INH/RFMP 3–4), and 3–4 months daily rifampin alone (RFMP 3–4) [2]. INH/RPT-3 is a relatively new regimen, the simplicity and short duration of which offer considerable appeal. The PREVENT TB trial, a large randomized controlled trial (RCT) of INH/RPT-3, recently demonstrated non-inferiority to the standard LTBI treatment regimen of INH-9 [5]. A subsequent analysis focusing on the pediatric population within this trial found similar results [6].

Given the presence of multiple regimens from which patients’ treatment can be chosen, network meta-analysis is needed to compare their merits. A 2014 review including network meta-analyses evaluated the efficacy of 15 regimens for LTBI treatment [7]. They found that INH-6, INH 12–72 months (INH 12–72), RFMP 3–4, and INH/RFMP 3–4 were all superior to both placebo and pyrazinamide-containing regimens. However, 28 of 53 included studies included populations in which LTBI was not confirmed with either TST or IGRA. No gold standard exists for the diagnosis of LTBI and patients may have LTBI despite negative TST and IGRA [2]. Nonetheless, including studies without confirmed LTBI means that an unknown proportion of the patients may have been uninfected, complicating interpretation of their analysis. Thus, the applicability of these results to populations with confirmed LTBI is unclear. Further, analyses in the review did not account for variation in duration of follow-up across studies. Although the annual risk of TB reactivation decreases with time, the cumulative risk increases [8, 9]. This means that, with all else being equal, studies with longer follow-up would be expected to have more events, complicating between study comparisons.

While efficacy is an important consideration in regimen selection, other factors such as anticipated completion are also vital. A recent meta-analysis of studies including patients taking a variety of regimens found an aggregated completion rate of only 61% [10]. However, to our knowledge no review has compared completion rates between different LTBI treatment regimens.

To address these concerns, we performed a systematic review with network meta-analyses of studies including patients with confirmed LTBI to assess whether the INH/RPT-3 regimen had greater rates of efficacy and completion compared to INH-9, INH-6, INH/RFMP 3–4, and RFMP-4 regimens.

## Methods

A review protocol was drafted prior to initiation of the review and is available upon request from the authors.

### Searching the literature

The aforementioned 2014 review served as the starting point for identification of studies for the current review [7]. An expanded search was developed by an information specialist; strategies combined controlled vocabulary and keywords and were reviewed prior to execution by a senior information specialist using the Peer Review for Electronic Search Strategies checklist [11]. Vocabulary and syntax were adjusted across databases. We searched MEDLINE, Embase, CENTRAL, PubMed, ICTRP, and additional sources including elements of the grey literature. Studies published up to June 2016 were included. Details of the search are presented in the review’s supplemental information (see Additional file 1).

### Study selection

Studies were included if they were RCTs involving patients of any age and reporting on efficacy (i.e. prevention of active TB) or completion rates of at least one of the regimens of primary interest (INH/RPT-3, INH-9, INH-6, INH/RFMP3–4, and RFMP-4). Regimens containing PZA were not considered among those of primary interest due to their poor toxicity profile [12]. Other regimens were included as sources of indirect evidence, namely placebo, no treatment, INH 3–4 months, INH 12–72 months, RFMP/PZA-2, and INH/RFMP/PZA-3. The study population had to consist of patients diagnosed with LTBI by positive TST and/or IGRA. Studies of patients without confirmed LTBI and studies with mixed confirmed and unconfirmed populations were excluded. Publications in non-English languages were included and assessed by a reviewer fluent in that language. Titles and abstracts from the primary search were independently assessed by 2 reviewers. Those seemingly meeting inclusion criteria were further assessed by review of full texts by the same 2 reviewers. Disagreements were resolved by consensus. The process of study selection was documented in a flow diagram (see Additional file 1).

### Data extraction and risk of bias assessment

Data were extracted by one of two reviewers and were checked for accuracy by a third reviewer. Extracted data included publication traits (e.g. year, study design, country, funding), population characteristics (e.g. patient age, sex, ethnicity, comorbidities, and risk factors), intervention details, outcomes (incidence of TB, treatment completion), and design (follow-up, randomization, blinding, allocation concealment). Risk of bias was assessed using the Cochrane Collaboration’s Risk of Bias Tool [13]. A narrative summary of assessments was compiled to identify variations in the risk of bias across studies.

### Structure of evidence networks

For efficacy, nodes in the treatment network were taken to be the specific regimens identified from the included studies: placebo, no treatment, INH/RPT-3, INH-9, INH-6, INH/RFMP3–4, RFMP-4, INH 3–4, INH 12–72, RFMP/PZA-2, and INH/RFMP/PZA-3. Placebo and no treatment were considered equivalent.

For treatment completion, we anticipated that the percentages of patients completing shorter treatment regimens were likely to be generally greater than the corresponding proportions of patients consuming longer treatment regimens. Given this expectation, it was felt that, to maximize homogeneity within nodes in the evidence network, placebo groups of different duration should be considered as individual nodes rather than collectively grouped. Thus, we considered placebo for durations of 3 months (placebo-3), 6 months (placebo-6), 9 months (placebo-9), and > = 12 months (placebo-12) to be distinct interventions.

### Methods for evidence synthesis

Comparisons of efficacy and treatment completion between regimens were estimated using network meta-analysis (NMA). Both fixed effects (FE) and random effects models with a vague prior distribution for between study variance (from here onward called *RE vague*; specifically, Uniform(0,5)) were planned. Vague prior distributions for treatment effects (i.e. Normal(0, 10,000)) were used for all analyses. Given the high prevalence of single study connections in both treatment networks, RE models using an informative prior distribution for the between study variance (from here onward called *RE informative*) were also performed to provide more realistic estimates of the between study variance than could be estimated from the sample data alone. Priors used for between study variance in these analyses were chosen based on empirical estimates previously reported elsewhere [14], specifically lognormal(μ = −3.23, σ^2^ = 1.88^2^); as model fit was adequate for both endpoints and comparable to RE vague fit based upon deviance information criteria, these results were chosen to be the basis for primary clinical interpretations. To summarize evidence from head-to-head trials and inspect levels of statistical heterogeneity within the network, we performed traditional pairwise meta-analyses prior to the NMAs. NMAs were performed using established models described elsewhere [15–17]. Efficacy was analyzed using a Poisson model for NMA, while completion was analyzed using a model for binary endpoints. We present summary estimates from efficacy analyses as rate ratios, while summary estimates for comparison of completion are reported as odds ratios; both are reported with 95% credible intervals (CrI). Surface Under the Cumulative Ranking curve (SUCRA) values per intervention were also estimated [18]; SUCRA values range between 0 and 1, with values nearer 1 indicative of a preferred treatment. Further details regarding methods for NMA (models used, assessment of model fit, evaluation of model convergence, checks for inconsistency and software) are provided in the review’s supplement (see Additional file 1). Reporting of findings was guided by the PRISMA Extension Statement for NMA [19].

Efficacy analyses were based on the reported numbers of confirmed and probable cases of TB in each study; case definitions are provided in the review’s supplemental information (see Additional file 1). To account for differences in follow-up across studies, efficacy was analyzed using a model for rates based on the number of cases in intervention groups and the corresponding person years followed [15]. Completion was analyzed as a binary endpoint using an established model wherein the primary analysis included all studies wherein completion was defined within trials to require between 80 and 100% medication consumption.

Sensitivity analyses related to population characteristics including average patient age, year of publication, and presence of comorbidities (including HIV infection, history of transplant or silicosis) were also performed for efficacy using meta-regression. Efficacy was also analyzed as a binary endpoint in a supplemental analysis. Additional analyses of the completion endpoint included (i) restriction of the range of completion criteria to 80–90%; and (ii) inclusion of additional studies from the review by Stagg et al. [7] which were not conducted explicitly in LTBI patients, with the rationale that presence or absence of a confirmed LTBI diagnosis would not impact completion.

## Results

### Study characteristics

A flow diagram provided in the current review’s supplement (see Additional file 1) summarizes the process of study selection. A total of 35 publications describing 30 unique studies initially met eligibility criteria [5, 6, 20–52]. Totals of 16 studies (*n* = 44,149) [5, 20, 21, 23, 26, 29, 31, 32, 37, 41–44, 46, 48, 49] and 14 studies (*n* = 44,128) [5, 20, 26, 27, 29, 31–33, 37, 41–44, 46] studies were included in the efficacy and completion NMAs, respectively. Thirteen studies met eligibility criteria but were judged to be heterogeneous related to aspects of endpoint definition, overlap of patients enrolled, treatment comparisons (which did not align with the network structure), and differences in patient population [6, 23–25, 30, 34, 36, 39, 45, 47, 50–52]; a section of the review’s supplemental information (see Additional file 1) details these studies and their findings.

Nine different regimens were used in the included RCTs. Table 1 summarizes characteristics of the 30 RCTs, while a detailed summary of study-specific information is provided in the review’s supplemental information (see Additional file 1). Studies were published between the years 1968–2016 (median 2005), and were associated with a median sample size of 353 participants (range 37–27,830). The mixture of geographic locations of studies was broad. The median value of average patient age across studies was 34.7 (range 3.6–59.7), and 2 RCTs [30, 45] enrolled only children (a companion article for one study also present data in children [6]). Five studies were conducted strictly in HIV-infected patients, [26, 37, 41–43] 1 in transplant patients [21], 2 in patients with silicosis [35, 44], and 2 in prisoners [24, 25]. Regarding outcomes, completion criteria were variably defined as consumption of either >80%, >90%, 95% or 100% of doses. While treatments in most studies were self-administered, 5 involved directly observed therapy [5, 6, 24, 25, 43]. In all studies, INH/RPT-3 administration was directly observed.

**Table 1:** Overview of characteristics of included randomized trials

Characteristic	Summary measure
Study sample size	
Median (range)	352 (37–27,830)
Year of publication (median, range)	Median 2005 (range 1968–2016)
Before 1980	3 (10%)
1981–1990	1 (3.3%)
1991–2000	6 (20%)
2001–2010	10 (33.3%)
2011–2016	10 (33.3%)
% Female participants	
Median (range)	45.5% (0%–83.3%)
Average patient age (years)	
# studies reporting mean/median	23
# with average age between <20	3 (13.0%)
# with average age between 20 and 40	13 (56.5%)
# with average age > 40	7 (30.5%)
Other population characteristics of note	
# enrolling HIV patients	5 (17.2%)
# in prison populations	2 (6.9%)
# in population at risk of silicosis	3 (10.3%)
# in transplant patients	1 (3.5%)
Funding source	
Industry	3 (10%)
Academic/government	15 (50%)
Mixed funding	1 (3.3%)
Not reported	11 (36.7%)

### Risk of bias assessments of the included RCTs

Details of the study-specific risk of bias evaluations are provided in the online supplement (see Additional file 1). Reporting of methods for randomization and allocation concealment was limited; most studies were judged unclear for risk of selection bias. Regarding risk of performance bias due to attrition or non-blinding of patients, personnel or outcome assessors, many studies provided limited or no relevant information, while others reported being open-label. Only two studies were judged at low risk of bias. Reporting bias mostly could not be assessed due to a lack of access to protocols for older studies. For six studies with a protocol available, all were found to demonstrate consistency in data reported. Regarding assessment efficacy, totals of 3 and 2 studies of the 16 with available data were judged to be of high and unclear risk of bias, respectively, while the remaining 11 were judged as low risk of bias; judgments of high risk of bias were related to the potential for disease status misclassification due to methods for diagnosis of active TB, while judgments of unclear were related to a failure to report the means of diagnostic testing. The remaining content of the main text of the review is focused upon studies retained for inclusion in NMAs.

#### Interventions represented in treatment networks

**Fig. 1:**
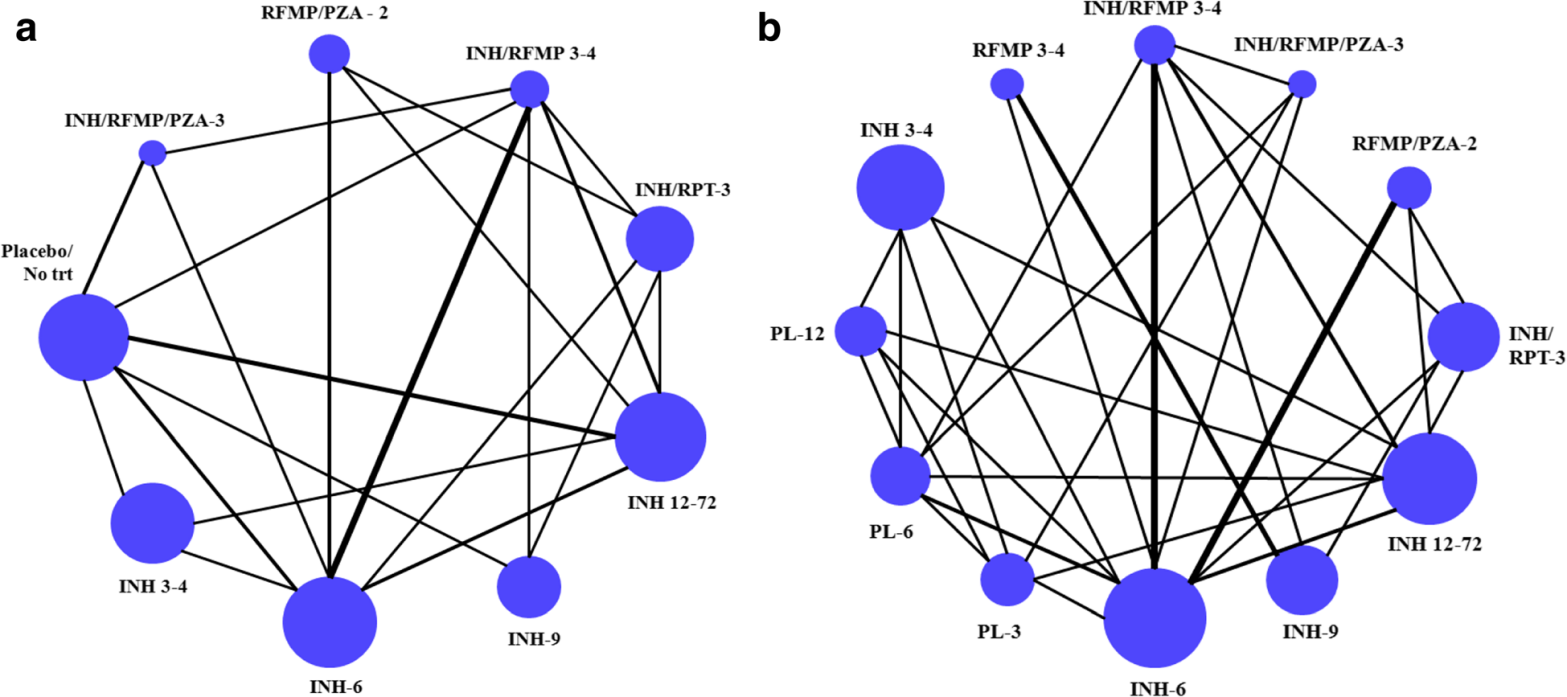
**a** and **b**: Network Diagrams, Available Evidence for Efficacy (Panel **a**) and Completion (Panel **b**). Totals of 16 RCTs (44,149 participants) and 14 RCTs (44,128 participants) were available for analyses of efficacy and completion, respectively. Treatment nodes are sized to reflect the proportion of patients studied on each intervention relative to the total number of patients studied. Edges joining different interventions are sized to reflect the proportion of studies informing each comparison (minimum 1 study). In comparisons where there is no line adjoining a pair of nodes, no eligible trials were identified. The online supplement provides a detailed summary of the numbers of studies in each connection as well as the total number of patients randomized to each intervention. Abbreviations. INH = isoniazid; RPT = rifapentine; RFMP = rifampin; PZA = pyrazinamide; trt = treatment; PL = placebo

Panels A and B of Fig. 1 present network diagrams for analyses of efficacy and regimen completion. Amongst the 16 RCTs [5, 20, 21, 23, 26, 29, 31, 32, 37, 41–44, 46, 48, 49] (*n* = 44,149) analyzed for efficacy, data from head-to-head trials were available for 21/36 (58.3%) of the possible pairwise comparisons in the network, with single studies informing several of the comparisons. Of the 14 RCTs (*n* = 44,128) used for the NMA of completion [5, 20, 26, 27, 29, 31–33, 37, 41–44, 46], head-to-head trials were available for 30/66 (45.5%) of the possible pairwise comparisons. The criteria used across studies to meet the endpoint of regimen completion ranged between 80 and 100% for the primary analysis. There were several studies for regimens involving INH mono-therapies available, while comparisons involving other regimens often were informed by only one or two trials.

#### Findings, efficacy

**Fig. 2:**
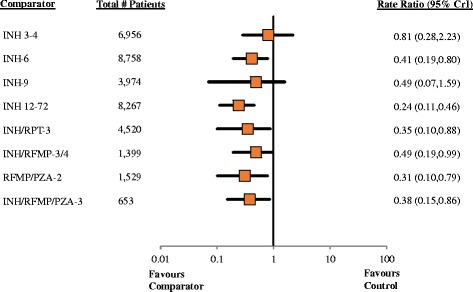
Efficacy, Pairwise Comparisons versus Placebo From Network Meta-Analysis. Pairwise comparisons from the RE informative analysis are shown as rate ratios and 95% CrIs, focusing on comparisons of active comparators versus control (placebo/no treatment) in the network. Values <1 suggest additional benefit with the comparator. A league table of all summary comparisons from the analysis is provided in Fig. 3

**Fig. 3:**
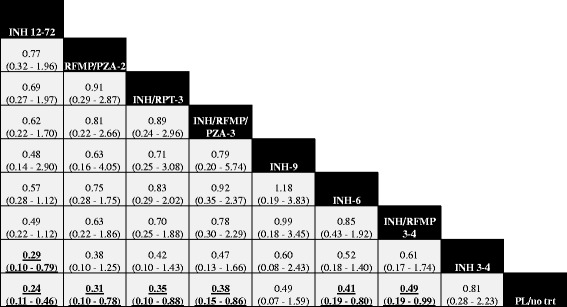
Summary of Findings from RE Informative Prior Network Meta-Analysis, Efficacy (Rate Ratios with 95% CrI). A complete summary of estimates from the RE informative network meta-analysis for efficacy is shown. Statistically significant differences between regimens are shown in bold, underlined font. Treatments are ordered from upper left to lower right in order of decreasing SUCRA value. To draw interpretations from the results, the lower/right-most comparison for each comparison is the reference treatment. Abbreviations. INH = isoniazid; RPT = rifapentine; RFMP = rifampin; PZA = pyrazinamide

Fit of the RE informative and RE vague models were both adequate, while fit of the FE model was limited (Table 2). Rate ratios from the RE informative NMA summarizing comparisons of active regimens versus the control group are presented in Fig. 2 (analogous estimates from the RE vague and FE models are provided in Table 2). Overall, findings from the RE informative model suggested a lower rate of active TB with each of the active regimens than observed with the control group, with benefits reaching statistical significance for all but INH-9 and INH 3–4. Comparisons between active regimens from the RE informative model are summarized in the league table presented in Fig. 3; no statistically significant differences between regimens were found. Table 2 summarizes estimates of effect versus control from each of the RE informative, RE vague and FE analyses along with SUCRA values associated with each treatment regimen. Summary estimates from the RE vague model were associated with similar effect estimates but wider 95% credible intervals, while findings from the FE model had narrower credible intervals but warrant more cautious interpretation based on limitations of model fit. League tables providing full summaries of findings from the RE vague and FE analyses are provided in the supplement (see Additional file 1).

**Table 2:** Summary of findings from network meta-analysis across models

Intervention	RE informative analysis		RE vague analysis		FE analysis	
	RR (95% CrI)	SUCRA	RR (95% CrI)	SUCRA	RR (95% CrI)	SUCRA
Treatment efficacy						
Control (reference trt)	1	0.06	1	0.08	1	0.03
INH 3–4	0.81 (0.28–2.23)	0.17	0.80 (0.18–3.29)	0.20	0.82 (0.61–1.09)	0.15
INH-6	*0.41 (0.19–0.80)*	0.52	*0.40 (0.14–1.00)*	0.50	*0.42 (0.32–0.55)*	0.56
INH-9	0.49 (0.07–1.59)	0.46	0.36 (0.03–1.67)	0.56	0.62 (0.26–1.46)	0.31
INH12–72	*0.24 (0.11–0.46)*	0.89	*0.22 (0.08–0.54)*	0.84	*0.24 (0.17–0.34)*	0.97
INH/RPT-3	*0.35 (0.10–0.88)*	0.67	0.31 (0.07–1.11)	0.65	*0.37 (0.21–0.61)*	0.68
INH/RFMP 3–4	*0.49 (0.19–0.99)*	0.42	0.45 (0.13–1.18)	0.44	*0.52 (0.36–0.75)*	0.38
RFMP/PZA-2	*0.31 (0.10–0.78)*	0.72	0.29 (0.06–1.01)	0.68	*0.32 (0.19–0.52)*	0.79
INH/RFMP/PZA-3	*0.38 (0.15–0.86)*	0.59	0.36 (0.11–1.12)	0.56	*0.39 (0.24–0.61)*	0.63
Resdev; # DP	41.92; 38		38.0; 38		57.3; 38	
DIC	203.88		200.9		213.2	
SD	0.44 (0.02–1.06)		0.66 (0.25–1.49)		NA	
Treatment completion						
Intervention	OR (95% CrI)	SUCRA	OR (95% CrI)	SUCRA	OR (95% CrI)	SUCRA
Placebo-12 (reference trt)	1	0.06	1	0.14	1	0.08
Placebo-3	*4.17 (1.96–8.60)*	0.88	*4.15 (1.64–10.45)*	0.76	*4.44 (3.78–5.23)*	0.88
INH-3/4	*3.01 (1.39–6.42)*	0.68	*3.01 (1.15–7.94)*	0.68	*3.01 (2.68–3.36)*	0.68
INH/RPT-3	*3.58 (1.40–8.83)*	0.79	*3.54 (1.17–10.44)*	0.84	*3.87 (2.56–5.80)*	0.95
RFMP/PZA-2	*2.44 (1.11–5.36)*	0.54	2.45 (0.94–6.50)	0.58	*2.28 (1.87–2.77)*	0.44
INH/RFMP/PZA-3	*2.36 (1.02–5.40)*	0.52	2.35 (0.85–6.64)	0.54	*2.41 (1.79–3.26)*	0.48
INH/RFMP 3–4	*3.14 (1.43–6.77)*	0.72	*3.12 (1.22–8.13)*	0.78	*3.20 (2.44–4.24)*	0.79
RFMP 3–4	*3.95 (1.15–13.72)*	0.81	3.95 (0.93–17.45)	0.79	*4.14 (2.49–6.87)*	0.89
Placebo-6	1.94 (0.95–3.88)	0.38	1.93 (0.80–4.67)	0.41	*2.00 (1.75–2.29)*	0.30
INH-6	1.49 (0.73–2.89)	0.22	1.48 (0.62–3.44)	0.32	*1.58 (1.42–1.75)*	0.18
INH-9	1.64 (0.57–4.45)	0.29	1.61 (0.46–5.38)	0.31	*1.87 (1.23–2.83)*	0.43
INH12–72	1.16 (0.59–2.45)	0.11	1.19 (0.52–3.03)	0.07	0.97 (0.87–1.07)	0.01
Resdev; # DP	36.33; 35		35.22; 35		63.93; 35	
DIC	274.54		274.06		295.29	
SD	0.33 (0.16–0.63)		0.41 (0.20–0.83)		NA	

Pairwise comparisons versus the reference treatment estimated from network meta-analysis are shown for treatment efficacy and treatment completion. Comparisons from three analyses (RE vague prior, RE informative prior, and FE) are presented as rate ratios (RR) for the efficacy analysis and odds ratios (OR) for the completion analysis, respectively, along with 95% credible intervals. Pairwise comparisons shown in italic font represent statistically significant differences between interventions. ﻿ SUCRA values are reported alongside each intervention. Measures of model fit are also provided for each analysis

Details of findings from sensitivity analyses for efficacy are also provided in the online supplement (see Additional file 1). Briefly, univariate meta-regression analyses were performed adjusting for average patient age, year of study publication, presence of HIV infection, presence of silicosis and history of transplantation in study populations. These analyses found interpretations of effect estimates were relatively unchanged. A supplemental analysis treating the endpoint as binary also showed little change in the ordering of treatment ranks and interpretations drawn.

#### Findings, completion of treatment

Assessment of treatment adherence varied between studies. Three studies used a combination of urinary testing, patient self-report and pill counts [20, 37, 42]. An additional 5 studies also used pill counts alone or combined with self-report [29, 32, 44, 46, 52]. One study compared directly observed to self-administered INH/RPT-3 with compliance to the latter assessed by self-report [52]. In the other 3 studies involving INH/RPT-3, all doses of INH/RPT-3 were directly observed while adherence to the comparator regimen was assessed by self-report [5, 26, 31]. One additional study which compared INH-based regimens utilized directly observed therapy in all arms [36]. One study utilized attendance at weekly visits [43] and the remaining two studies used an electronic device that measured the timing of pill bottle opening [27, 33].

**Fig. 4:**
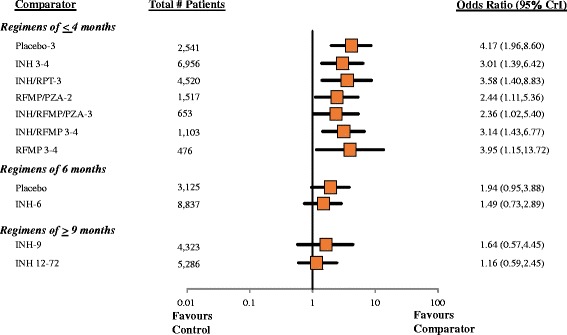
Forest Plot, Comparisons versus Placebo-12 from Network Meta-Analysis, Completion. Pairwise comparisons from the RE informative anlaysis are shown as summary odds ratios and 95% CrIs, focusing on comparisons of active comparators versus the control group of Placebo-12 months in the network. Values >1 suggest greater likelihood of completion with the comparator, and regimens have been grouped according to duration. A league table of all summary comparisons from network meta-analysis is provided in Fig. 5

**Fig. 5:**
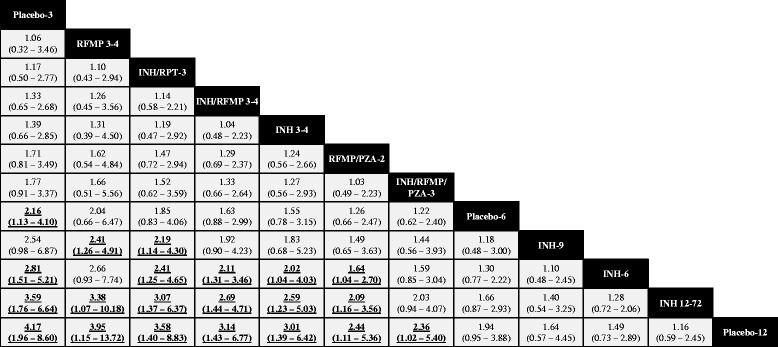
Summary of Findings from RE Informative Prior Network Meta-Analysis (Odds Ratios with 95% CrI),. Completion of Treatment. A complete summary of estimates from the RE informative analysis for treatment completion is provided. Statistically significant differences between regimens are shown in bold, underlined font. Treatments are ordered from upper left to lower right in order of decreasing SUCRA value from the random effects analysis. To draw interpretations from the results, the lower/right-most comparison for each comparison is the reference treatment. *Abbreviations*. INH = isoniazid; RPT = rifapentine; RFMP = rifampin; PZA = pyrazinamide

Fit of the RE informative and RE vague models was again adequate, while fit of the FE model was limited (Table 2). Figure 4 presents odds ratios from the RE informative analysis comparing treatment completion of different regimens to the chosen reference treatment, Placebo-12; Fig. 5 presents a league table summarizing all pairwise comparisons. Overall, regimens of shorter duration were more likely to demonstrate higher completion rates than those of longer duration. Evidence for improved completion was strongest for shorter rifamycin-based regimens relative to other regimens of 6 months and longer. Several comparisons from the RE informative analysis demonstrated benefits relative to regimens of longer duration. Table 2 summarizes estimates of effect versus control from each of the RE informative, RE vague and FE analyses along with SUCRA values associated with each treatment regimen. Patterns observed mirrored those described for the efficacy analysis in terms of similarity of effect estimates and width of credible intervals.

Findings from sensitivity analyses are presented in the review’s online supplement (see Additional file 1). First, an additional analysis to restrict accepted definitions of treatment completion from 80 to 100% to 80–90% was performed (11 studies, 12 treatments, 39,410 patients); summary estimates of effects from the primary analysis remained relatively unchanged. Second, a supplemental analysis was performed wherein studies from the existing review [7] which did not have formal criteria for LTBI infection were also added to the network (an additional 11 studies, and 64,819 patients). Results were associated with increased precision and similar findings in terms of differences between therapies; additionally, the estimate comparing INH-6 with INH-9 changed to indicate a greater likelihood of completion with the former, a more intuitive result than observed in the primary analysis (though in both cases this difference was not statistically significant).

Model fit results for all analyses are provided throughout various portions the review’s main text (Table 2) and online supplement (see Additional file 1). Assessment of DIC across for the primary analyses based on RE informative models did not suggest evidence of inconsistency in the analyses.

## Discussion

We performed a systematic review with network meta-analyses with an interest in comparing the efficacy and completion rates of INH/RPT-3 compared to INH-9, INH-6, INH/RFMP-3/4, and RFMP-4. Other regimens and inactive control groups were also included as sources of indirect evidence. All regimens of a priori interest except INH-9 showed a statistically significant benefit in preventing active TB compared to placebo. Amongst regimens of primary interest for this review, RFMP 3–4, INH/RPT-3 and INH/RFMP 3–4 were associated with higher rates of completion than 12 months of placebo but INH-6 and INH-9 were not.

As both of the evidence networks analyzed in the current review were found to be comprised of many single study connections, there was concern that the limited available data might not provide realistic estimates of the between study variance parameter if using a vague prior distribution for this parameter, as is common in a standard random effects analysis. We thus post-hoc felt it important to perform additional random effects analyses using an informative prior distribution chosen in consideration of empirical estimates [14] which were felt to be reasonable choices for the current review. As it was felt that findings from this analysis provided the best approach to manage concerns regarding variance estimates while respecting heterogeneity amongst studies, it was judged by the research team to be the most representative analysis of the data. Findings from the RE vague and FE analyses have also been reported to provide readers with findings from all three analytic approaches for transparency.

A 2014 review [7] used network meta-analysis to assess the relative efficacy of LTBI treatments. The efficacy analysis in the current review differs by including only studies in which patients had confirmed LTBI, by accounting for differences in follow-up by using rate ratios to assess efficacy, and by performing an additional NMA of completion rates. Our criteria eliminated 28 studies included in the previous meta-analysis. Trials not confirming LTBI likely contained an unknown number of patients without infection, potentially impacting the apparent treatment effect and precluding accurate comparisons across studies. By excluding such trials, this review may provide a more focused portrait of regimen efficacy. A notable difference in the findings of the two studies is that the present study found a statistically significant benefit for the INH/RPT-3 regimen while the prior NMA did not.

Although INH-9 is widely recommended as first line therapy for LTBI [2, 12], this treatment showed low efficacy among the regimens of interest and was not found to be significantly more efficacious than placebo. The recommendation for INH-9 is based on a re-analysis and extrapolation of data from trials conducted in the 1950s–60s, and few trials have included this regimen [53]. This paucity of trials may have contributed to uncertainty in the efficacy estimate for this regimen in our study. Further, variation in rates of development of active TB was noted between trials. In general, trials including INH-9 had low rates of active TB in all arms, suggesting that their populations may have had a lower baseline risk of TB reactivation and hence a lesser opportunity for benefit from treatment. This could lead to an apparent lack of benefit for this regimen when compared to regimens tested in higher risk populations where greater absolute reductions in TB reactivation were observed. Populations studied may have, to some degree, varied in their baseline risk of reactivation.

Anticipated compliance is a key factor in treatment selection since poor compliance would be expected to lessen potential benefit. Despite this, data on comparative rates of completion remain limited; this area has been highlighted by the WHO as an important research gap [2]. To our knowledge, this is the first NMA comparing completion rates between LTBI treatment regimens. Results from this review demonstrate an overall pattern toward improved completion with shorter regimens. A sensitivity analysis including studies in which LTBI was not confirmed showed a similar trend.

While it may seem intuitive that shorter duration regimens would be associated with higher compliance, other factors such as drug tolerability and dosing schedule (e.g. once weekly dosing) could influence completion rates, making this study’s finding an important one. Our primary analysis grouped studies defining completion as taking anywhere from 80 to 100% of doses. A secondary analysis restricted to studies using a criterion of between 80 and 90% showed broadly similar results. A complication in our analysis of completion rates came from heterogeneity in study populations. Completion rates are known to vary widely between patient groups, with low rates observed among marginalized populations including prisoners and relatively higher completion among those with HIV [2]. This may help to explain some of the variability between regimens in the rates of active TB that we noted.

A strength of our study is its rigorous methodology, with inclusion of only studies confirming LTBI. This did, however, reduce the number of studies and patients in our analyses. It also excluded several large studies including many of the early trials of INH in the Alaskan Inuit and a large recent trial of INH prophylaxis in South African miners [54–57]. However, it was felt that this was justified to ensure that included trials had comparable populations. Further, our study used rate ratios as our primary efficacy outcome measure, thus incorporating follow-up duration into between-treatment comparisons.

Our study has limitations. From the perspective of a population, the number of instances of TB reactivation, and thus the opportunity for transmission, depends not only on the efficacy and completion rate of treatment, but also, crucially, on the acceptance rate (i.e. the proportion of patients offered treatment who elect to start it). Acceptance rates for LTBI treatment are generally low, driving the ongoing TB burden in many parts of the world. Unfortunately, our identified studies did not report the rate of acceptance. Further, since all included trials were randomized, patients initially accepted the possibility of being assigned to any of several treatments rather than accepting a specific regimen. This precludes obtaining an acceptance rate that could be broadly applied outside of the clinical trial context. Additionally, the present study did not analyse differences in adverse events between treatments although the risk of such events is another factor influencing regimen selection. An additional limitation is that the effect of directly observing therapy (DOT) on completion rates could not be fully addressed. This was because few studies used DOT, with the exception that INH/RPT-3 was directly observed in all studies included in our analysis. This meant that data were sparse to compare the effect of DOT on completion for individual regimens.

## Conclusion

Shorter rifamycin-based regimens may offer comparable benefits to longer INH regimens. Analyses of completion suggest shorter regimens of 3–4 months duration offer greater completion than longer regimens.

## Additional file

Additional file 1: Appendix 1. Description of Approach to Literature Search. **Appendix 2.** Flow Diagram, Process of Study Selection. **Appendix 3.** Supplementary material regarding data extraction, case definitions and NMA structure. **Appendix 4.** WinBugs Code for Network Meta-Analyses. **Appendix 5.** Studies Excluded from NMA. **Appendix 6.** Detailed Summary of Study Characteristics. **Appendix 7.** Summary of Risk of Bias Assessments. **Appendix 8.** Numbers of Studies Per Comparison and Patients Per Treatment for Primary Analyses. **Appendix 9.** Summary of Results from Pairwise Meta-Analyses. **Appendix 10.** Results From Sensitivity Analyses. **Appendix 11.** Model Fit Results from Primary Network Meta-Analyses. **Appendix 12.** PRISMA NMA Checklist. **Appendix 13.** Reference list for appendices. (DOCX 1669 kb)

## Abbreviations

CrI: Credible Interval
FE: Fixed Effects
INH: Isoniazid
LTBI: Latent Tuberculosis Infection
NMA: Network Meta-Analysis
PZA: Pyrazinamide
RCT: Randomized Controlled Trial
RE: Random Effects
RFMP: Rifampicin
RPT: Rifapentine
SUCRA: Surface Under the Cumulative Ranking
TB: Tuberculosis
