# Supplementary material for: Efficacy and completion rates of rifapentine and isoniazid (3HP) compared to other treatment regimens for latent tuberculosis infection: a systematic review with network meta-analyses
Source: BMC Infect Dis. 2017 Apr 11;17:265. doi: 10.1186/s12879-017-2377-x (PMC5387294; doi:10.1186/s12879-017-2377-x)
Supplement: Supplementary file 1 — Description of Approach to Literature Search. Appendix 2. Flow Diagram, Process of Study Selection. Appendix 3. Supplementary material regarding data extraction, case definitions and NMA structure. Appendix 4. WinBugs Code for Network Meta-Analyses. Appendix 5. Studies Excluded from NMA. Appendix 6. Detailed Summary of Study Characteristics. Appendix 7. Summary of Risk of Bias Assessments. Appendix 8. Numbers of Studies Per Comparison and Patients Per Treatment for Primary Analyses. Appendix 9. Summary of Results from Pairwise Meta-Analyses. Appendix 10. Results From Sensitivity Analyses. Appendix 11. Model Fit Results from Primary Network Meta-Analyses. Appendix 12. PRISMA NMA Checklist. Appendix 13. Reference list for appendices. (DOCX 1669 kb) [file 12879_2017_2377_MOESM1_ESM.docx]

**Additional File 1:**

**Online Systematic Review Appendices**

*Efficacy and completion rates of rifapentine and isoniazid (3HP) compared to other treatment regimens for latent tuberculosis infection: a systematic review with network meta-analyses. Pease C, Hutton B, Yazdi F, Wolfe D, Hamel C, Quach P, Skidmore B, Moher D, Alvarez G.*

- **Appendix 1:** Description of Approach to Literature Search
- **Appendix 2:** Flow Diagram, Process of Study Selection
- **Appendix 3:** Supplementary material regarding data extraction, case definitions and NMA structure
- **Appendix 4:** WinBugs Code for Network Meta-Analyses
- **Appendix 5:** Studies Excluded from NMA
- **Appendix 6:** Detailed Summary of Study Characteristics
- **Appendix 7:** Summary of Risk of Bias Assessments
- **Appendix 8:** Numbers of Studies Per Comparison and Patients Per Treatment for Primary Analyses
- **Appendix 9:** Summary of Results from Pairwise Meta-Analyses
- **Appendix 10:** Results From Sensitivity Analyses
- **Appendix 11:** Model Fit Results from Primary Network Meta-Analyses
- **Appendix 12:** PRISMA NMA Checklist
- **Appendix 13:** Reference list for appendices

**Appendix 1: Description of Approach to Literature Search**

A 2014 systematic review by Stagg et al in the Annals of Internal Medicine served as the starting point for the identification of studies for the current review. From this an expanded search was developed. This had two objectives: (1) to identify new studies that have emerged since that publication; and (2) to also identify non-randomized studies of relevance for a detailed systematic review of harms associated with LTBI interventions (this work is currently in progress and will be the subject of a future publication).

The search strategy for this work was developed and tested through an iterative process by an experienced medical information specialist in consultation with the review team (Becky Skidmore). Using the OVID platform, we searched Ovid MEDLINE®, Ovid MEDLINE® In-Process & Other Non-Indexed Citations, and Embase. We also searched the CENTRAL database using the Cochrane Library on Wiley. The search was supplemented by a search of PubMed for publisher-supplied and recently added citations.

Strategies utilized a combination of controlled vocabulary (e.g., “Latent Tuberculosis”, “Chemoprevention”, “Antitubercular Agents”) and keywords (e.g., inactive tuberculosis, tuberculostatic, 9INH). Vocabulary and syntax were adjusted across databases. We used a validated randomized controlled trial filter as well as other filters to identify observational, post-marketing and safety studies. The core strategy was reviewed prior to execution by another senior information specialist using the Peer Review for Electronic Search Strategies checklist.^1^

We selectively searched websites listed in CADTH’s Grey Matters Light for grey literature. Additionally, we searched the WHO’s ICTRP Search Portal and Clinical Trials.Gov for completed clinical trials, and hand-searched the bibliographies of pertinent references.

Specific details regarding the strategies appear below.

**Literature Search Strategy for the Review**

Database: Ovid MEDLINE(R) In-Process & Other Non-Indexed Citations and Ovid MEDLINE(R) <1946 to Present>

Search Strategy:

--------------------------------------------------------------------------------

1 Latent Tuberculosis/

2 (laten* adj2 (tuberculosis or TB or TBI)).tw,kw.

3 ((inactive or noninfectious or non-infectious or uninfectious) adj2 (tuberculosis or TB or TBI)).tw,kw.

4 LTBI.tw,kw.

5 or/1-4 [LATENT TUBERCULOSIS]

6 Latent Tuberculosis/dt

7 exp Tuberculosis/pc

8 Chemoprevention/

9 (chemoprevent* or chemo-prevent* or chemoprophyla* or chemo-prophyla*).tw,kw.

10 exp Antitubercular Agents/

11 (antitubercular* or anti-tubercular* or tuberculostatic*).tw,kw.

12 Isoniazid/

13 (Amidon or Andrazide or Antimicina or Antituberkulosum or Armazid or Armazide or Atcotibine or Azuren).tw,kw.

14 (Bacillin or BP 5015 or CCRIS 351 or Cedin or Cemidon or Chemiazid or Chemidon or Cotinazin).tw,kw.

15 (Defonin or Diforin or Dinacrin or Ditubin or Dow-isoniazid or Ebidene or EINECS 200-214-6 or Eralon or Ertuban or Eutizon or Evalon).tw,kw.

16 (Fimalene or FSR 3 or Ftivazide or Gink or HIA or Hidranizil or Hidrasonil or Hidrulta or Hidrun or HSDB 1647 or Hycozid or Hydrazid or Hydrazide or Hyzyd).tw,kw.

17 (Ido-tebin or Idrazide dell'acido isonicotinico or Idrazil or IN-73 or INAH or INH or Inh-Burgthal or Inizid or Iscotin or Isidrina or Ismazide or Isobicina or Isocid or Isocidene or Isocotin or Isohydrazide or Isolyn or Isonerit or Isonex or Isoniazid or Isoniazide or Isonicid or Isonico or Isonicotan or Isonicotil).tw,kw.

18 (Isonicotinhydrazid or Isonicotinic acid hydrazide or sonicotinic Acid Vanillylidenehydrazide or Isonicotinic hydrazide or Isonicotinohydrazide or Isonicotinoyl hydrazide or Isonicotinoylhydrazine or Isonicotinsaeurehydrazid or Isonicotinyl hydrazide or Isonicotinylhydrazine).tw,kw.

19 (Isonide or Isonidrin or Isonikazid or Isonilex or Isonin or Isonindon or Isonirit or Isoniton or Isonizide or Isotamine or Isotebe or Isotebezid or Isotinyl or Isozid or Isozide or Isozyd).tw,kw.

20 (L 1945 or Laniazid or Mybasan or Neo-tizide or Neoteben or Neoxin or Neumandin or Nevin or Niadrin or Nicazide or Nicetal or Nicizina or Niconyl or Nicotibina or Nicotibine or Nicotisan or Nicozide or Nidaton or Nidrazid or Nikozid or Niplen or Nitadon or Niteban or Nitebannsc 9659 or NSC 9659 or Nydrazid or Nyscozid).tw,kw.

21 (Pelazid or Percin or Phthisen or Phthivazid or Phthivazide or Preparation 6424 or Pycazide or Pyreazid or Pyricidin or Pyrizidin or Raumanon or Razide or Retozide or Rifamate or Rimicid or Rimifon or Rimiphone or Rimitsid or RP-5015 or Stanozide or Tubazid or Tubazide or UNII-V83O1VOZ8L).tw,kw.

22 Rifampin/

23 (Archidyn or Arficin or Arzide or "Ba 41166/E" or Benemicin or CCRIS 551 or Dione 21-acetate or Doloresum or EINECS 236-312-0 or Eremfat or Fenampicin or HSDB 3181).tw,kw.

24 (L-5103 or Lepetit or NSC 113926 or "R/AMP" or RAMP or Rifa or Rifadin or Rifagen or Rifaldazin or Rifaldazine or Rifam or Rifamor or Rifampicin or Rifampicine or Rifampin).tw,kw.

25 (Rifamycin or Rifaprodin or Rifcin or Rifinah or Rifobac or Rifoldin or Rifoldine or Riforal or Rimactan or Rimactane or Rimactazid or Rimactizid or Rimazid or Sinerdol or Tubocin or UNII-VJT6J7R4TR).tw,kw.

26 (3HP or 9H or 3INH or 4INH or 6INH or 9INH or "3INH/RPT" or "RPT/INH" or "RPT-INH" or RPTINH or RMP or "RMP/INH" or "RMP-INH" or RMPINH or "RPT/3INH" or "INH/RPT" or "INH-RPT" or INHRPT or "INH/RMP" or "INH-RMP" or INHRMP).tw,kw.

27 or/6-26 (129910) [INTERVENTIONS/COMPARATORS]

28 5 and 27 (1506) [LTBI and INTERVENTIONS/COMPARATORS]

29 (controlled clinical trial or randomized controlled trial or pragmatic clinical trial).pt.

30 clinical trials as topic.sh.

31 (randomi#ed or randomly or RCT$1 or placebo*).tw.

32 ((singl* or doubl* or trebl* or tripl*) adj (mask* or blind* or dumm*)).tw.

33 trial.ti.

34 or/29-33

35 28 and 34 [RCTS]

36 exp Cohort Studies/

37 cohort$1.tw.

38 Retrospective Studies/

39 (longitudinal or prospective or retrospective).tw.

40 ((followup or follow-up) adj (study or studies)).tw.

41 Observational study.pt

42 (observation$2 adj (study or studies)).tw.

43 ((population or population-based) adj (study or studies or analys#s)).tw.

44 ((multidimensional or multi-dimensional) adj (study or studies)).tw.

45 Comparative Study.pt.

46 ((comparative or comparison) adj (study or studies)).tw.

47 Cross-Sectional Studies/

48 ((cross-sectional or frequency or prevalence) adj (analys#s or study or studies or survey$1)).tw.

49 or/36-48

50 28 and 49 [OBSERVATIONAL STUDIES]

51 exp Product Surveillance, Postmarketing/

52 ((drug or drugs) adj3 (surveillance* or monitor* or register$1 or registry or registries)).tw.

53 pharmacovigilan*.tw.

54 "Clinical Trial, Phase IV".pt.

55 (("phase 4" or "phase IV") adj3 (clinical trial$1 or evaluat*)).tw.

56 adverse drug reaction report*.tw.

57 or/51-56

58 28 and 57 [POSTMARKETING STUDIES]

59 35 or 50 or 58 [RCTS, OBSERV, POSTMARKETING STUDIES]

60 exp Animals/ not (exp Animals/ and Humans/)

61 59 not 60 [ANIMAL-ONLY REMOVED]

62 (comment or editorial or interview or news).pt.

63 (letter not (letter and randomized controlled trial)).pt.

64 61 not (62 or 63) [OPINION PIECES REMOVED]

65 exp "Drug-Related Side Effects and Adverse Reactions"/

66 exp Drug-Induced Liver Injury/

67 Psychoses, Substance-Induced/

68 exp Drug Hypersensitivity/

69 drug recalls/ or safety-based drug withdrawals/

70 Abnormalities, Drug-Induced/

71 (safe or adverse or adversely or undesirable or harm* or injurious or risk or risks or reaction* or complication* or poison* or warning* or recall* or withdrawn* or withdrawal* or death or deaths or fatal or fatality or fatalities).ti.

72 (side effect* or safety or unsafe or toxic or toxicit* or toxologic* or intoxication or noxious or tolerability or teratogen* or drug induced or chemically induced).tw,kw.

73 ((adverse or undesirable or harm* or toxic or injurious or serious or fatal) adj3 (effect* or reaction* or event* or outcome* or incident*)).tw,kw.

74 exp Antitubercular Agents/ae, po, to

75 Isoniazid/ae, po, to

76 Rifampin/ae, po, to

77 or/65-76

78 28 and 77 [SAFETY]

79 78 not 60 ANIMAL-ONLY REMOVED]

80 79 not (62 or 63) [OPINION PIECES REMOVED]

81 64 or 80 [ALL STUDY DESIGNS]

**Appendix 2: Flow Diagram, Process of Study Selection**

As noted in Appendix 1, a 2014 systematic review by Stagg et al published in the fall of 2014 in the Annals of Internal Medicine^1^ served as the starting point for the identification of studies for the current review. From here, an expanded search was developed with a start state of January 2014. This had two objectives: (1) to identify new studies that had emerged since the work of Stagg et al; and (2) to identify non-randomized studies of relevance for a detailed systematic review of harms associated with LTBI interventions (work on this review is in progress). The flow diagram below summarizes the process of study selection based on screening of the citations identified from our search for literature to address these goals.

**
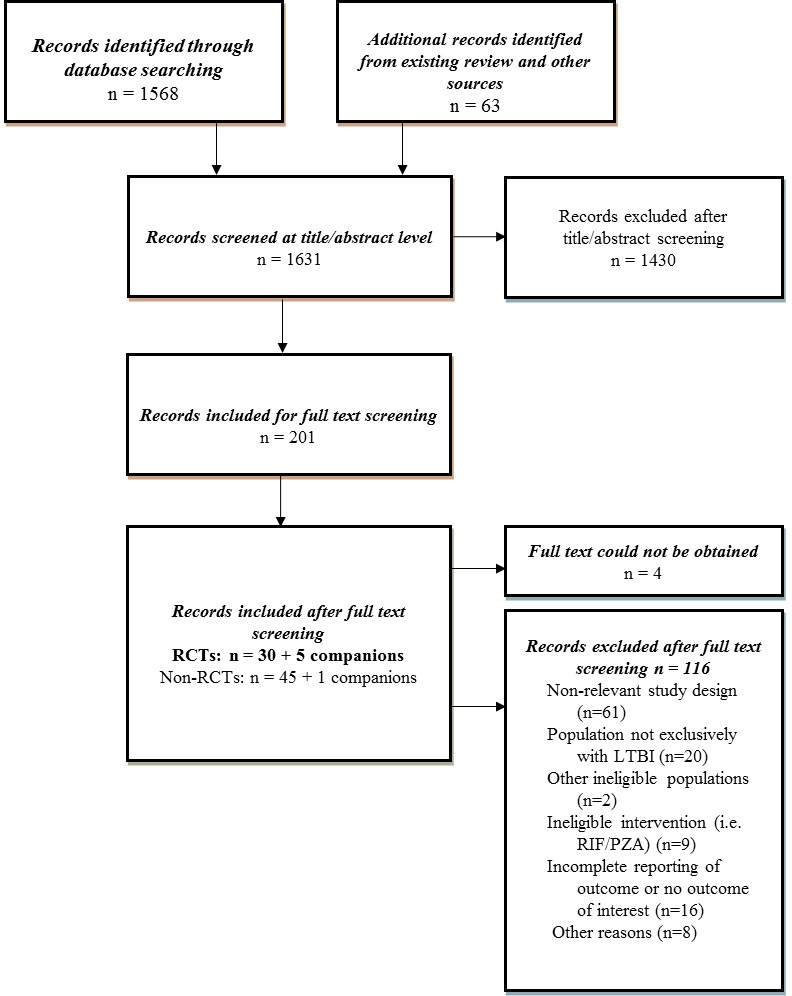
**

**Appendix 3:**

**Supplementary material regarding data extraction, case definitions and NMA structure**

***Data Extracted During the Review***

Extracted data included basic publication traits (year of publication, study design, country of the corresponding authors, duration of follow-up, funding source), population characteristics (e.g. patient age, gender, ethnicity, comorbidities, country of enrollment, and risk factors including incarceration, history of drug and alcohol use, smoking status and silicosis), intervention and comparators (e.g. drugs provided, including name, dose, duration and frequency of administration), outcomes (incidence of TB, treatment completion, and corresponding criteria per study), and study design (including measures of patient follow-up and description of other features including randomization, blinding and allocation concealment).

***TB cases Definitions***

Cases were considered confirmed if either culture positive for M. tuberculosis or acid-fast bacilli on tissue histology or sputum smear. Given the difficulty of obtaining sputa in pediatric studies, clinical criteria plus consistent radiographic findings were considered confirmatory. Probable cases were defined as those not fitting criteria for confirmed TB but suspected by radiographic appearance, response to anti-TB treatment or clinical suspicion.

***Structure of Evidence Networks and Methods for Network Meta-Analysis***

For analyses of efficacy, nodes in the treatment network were taken to be the specific regimens identified from the included studies: placebo, no treatment, INH/RPT-3, INH-9, INH-6, INH/RFMP3-4, RFMP-4, INH 3-4, INH 12-72, RFMP/PZA-2, and INH/RFMP/PZA-3. Placebo and no treatment were considered to be equivalent.

For treatment completion, representation of untreated placebo groups was modified to reflect differences in the duration of therapy (i.e. placebo in some studies was of shorter duration than others and thus likely easier for a larger proportion of patients to successfully complete). Thus, we considered placebo for durations of 3 months (placebo-3), 6 months (placebo-6), 9 months (placebo-9), and >=12 months (placebo-12) to be distinct interventions in data analyses.

Vague prior distributions were used for treatment effects of interest for all analyses presented in the report. Random effects and fixed effects analyses were performed as described in the main text of the review. Efficacy was analyzed using a Poisson model for network meta-analysis, while treatment completion was analyzed using a model for binary endpoints. Adequacy of model fit for all analyses was assessed by comparing posterior residual deviance to the number of unconstrained data points (i.e. number of intervention arms across studies) corresponding to each analysis, while comparison of fit between models was based upon the deviance information criteria (DIC) where a difference of 5 or more points was considered indicative of an important difference. All analyses were based on burn-in and sampling iterations of 40,000 or more. Model convergence was assessed by assessment of Gelman-Rubin diagnostic plots and inspection of Monte Carlo standard error of all parameter estimates. The assumption of consistency was assessed using by fitting of inconsistency models for both endpoints and comparison of DIC with the corresponding consistency models. Analyses were performed using WinBugs (version 1.4.3, Cambridge, UK) and NetMetaXL (version 1.6.1, www.netmetaxl.com).^2^ Due to fewer than 10 studies per connection, funnel plots exploring for publication bias were not pursued.

**Appendix 4: WinBugs Code for Network Meta-Analyses**

Code made available for RE vague and FE analyses from the NICE Technical Support Series for binary endpoints and rate endpoints used in the review are provided below. For analyses involving informative prior distributions, code was modified to use a lognormal distribution [lognormal(-3.23, 1.88^2^)] as suggested for semi-objective endpoints for comparison of pharmacologic interventions.

**RE Vague Binary**

# Binomial likelihood, logit link

# Random effects model for multi-arm trials

model{ # *** PROGRAM STARTS

for(i in 1:ns){ # LOOP THROUGH STUDIES

w[i,1] <- 0 # adjustment for multi-arm trials is zero for control arm

delta[i,1] <- 0 # treatment effect is zero for control arm

mu[i] ~ dnorm(0,.0001) # vague priors for all trial baselines

for (k in 1:na[i]) { # LOOP THROUGH ARMS

r[i,k] ~ dbin(p[i,k],n[i,k]) # binomial likelihood

logit(p[i,k]) <- mu[i] + delta[i,k] # model for linear predictor

rhat[i,k] <- p[i,k] * n[i,k] # expected value of the numerators

dev[i,k] <- 2 * (r[i,k] * (log(r[i,k])-log(rhat[i,k])) #Deviance contribution

+ (n[i,k]-r[i,k]) * (log(n[i,k]-r[i,k]) - log(n[i,k]-rhat[i,k])))

}

resdev[i] <- sum(dev[i,1:na[i]]) # summed residual deviance contribution for this trial

for (k in 2:na[i]) { # LOOP THROUGH ARMS

delta[i,k] ~ dnorm(md[i,k],taud[i,k]) # trial-specific LOR distributions

md[i,k] <- d[t[i,k]] - d[t[i,1]] + sw[i,k] # mean of LOR distributions (with multi-arm trial correction)

taud[i,k] <- tau *2*(k-1)/k # precision of LOR distributions (with multi-arm trial correction)

w[i,k] <- (delta[i,k] - d[t[i,k]] + d[t[i,1]]) # adjustment for multi-arm RCTs

sw[i,k] <- sum(w[i,1:k-1])/(k-1) # cumulative adjustment for multi-arm trials

}

}

totresdev <- sum(resdev[]) #Total Residual Deviance

d[1] <- 0 # treatment effect is zero for reference treatment

for (k in 2:nt){ d[k] ~ dnorm(0,.0001) } # vague priors for treatment effects

sd ~ dunif(0,5) # vague prior for between-trial SD. ALTERNATIVES BELOW

tau <- pow(sd,-2) # between-trial precision = (1/between-trial variance)

# pairwise ORs and LORs for all possible pair-wise comparisons, if nt>2

for (c in 1:(nt-1)) {

for (k in (c+1):nt) {

or[c,k] <- exp(d[k] - d[c])

lor[c,k] <- (d[k]-d[c])

}

}

} # *** PROGRAM ENDS

**FE Binary:**

# Binomial likelihood, logit link

# Fixed effects model

model{ # *** PROGRAM STARTS

for(i in 1:ns){ # LOOP THROUGH STUDIES

mu[i] ~ dnorm(0,.0001) # vague priors for all trial baselines

for (k in 1:na[i]) { # LOOP THROUGH ARMS

r[i,k] ~ dbin(p[i,k],n[i,k]) # binomial likelihood

logit(p[i,k]) <- mu[i] + d[t[i,k]] - d[t[i,1]] # model for linear predictor

rhat[i,k] <- p[i,k] * n[i,k] # expected value of the numerators

dev[i,k] <- 2 * (r[i,k] * (log(r[i,k])-log(rhat[i,k])) #Deviance contribution

+ (n[i,k]-r[i,k]) * (log(n[i,k]-r[i,k]) - log(n[i,k]-rhat[i,k])))

}

resdev[i] <- sum(dev[i,1:na[i]]) # summed residual deviance contribution for this trial

}

totresdev <- sum(resdev[]) #Total Residual Deviance

d[1]<-0 # treatment effect is zero for reference treatment

for (k in 2:nt){ d[k] ~ dnorm(0,.0001) } # vague priors for treatment effects

for (c in 1:(nt-1)) {

for (k in (c+1):nt) {

or[c,k] <- exp(d[k] - d[c])

lor[c,k] <- (d[k]-d[c])

} }

} # *** PROGRAM ENDS

**RE Vague Rates**

# Binomial likelihood, cloglog link

# Random effects model for multi-arm trials

model{ # *** PROGRAM STARTS

for(i in 1:ns){ # LOOP THROUGH STUDIES

w[i,1] <- 0 # adjustment for multi-arm trials is zero for control arm

delta[i,1] <- 0 # treatment effect is zero for control arm

mu[i] ~ dnorm(0,.0001) # vague priors for all trial baselines

for (k in 1:na[i]) { # LOOP THROUGH ARMS

r[i,k] ~ dbin(p[i,k],n[i,k]) # Binomial likelihood

cloglog(p[i,k]) <- log(time[i]) + mu[i] + delta[i,k] # model for linear predictor

rhat[i,k] <- p[i,k] * n[i,k] # expected value of the numerators

dev[i,k] <- 2 * (r[i,k] * (log(r[i,k])-log(rhat[i,k]))

+ (n[i,k]-r[i,k]) * (log(n[i,k]-r[i,k]) - log(n[i,k]-rhat[i,k]))) #Deviance contribution

}

resdev[i] <- sum(dev[i,1:na[i]]) # summed residual deviance contribution for this trial

for (k in 2:na[i]) { # LOOP THROUGH ARMS

delta[i,k] ~ dnorm(md[i,k],taud[i,k]) # trial-specific LOR distributions

md[i,k] <- d[t[i,k]] - d[t[i,1]] + sw[i,k] # mean of LOR distributions (with multi-arm correction)

taud[i,k] <- tau *2*(k-1)/k # precision of LOR distributions (with multi-arm correction)

w[i,k] <- (delta[i,k] - d[t[i,k]] + d[t[i,1]]) # adjustment for multi-arm RCTs

sw[i,k] <- sum(w[i,1:k-1])/(k-1) # cumulative adjustment for multi-arm trials

}

}

totresdev <- sum(resdev[]) #Total Residual Deviance

d[1]<-0 # treatment effect is zero for reference treatment

for (k in 2:nt){ d[k] ~ dnorm(0,.0001) } # vague priors for treatment effects

sd ~ dunif(0,5) # vague prior for between-trial SD

tau <- pow(sd,-2) # between-trial precision = (1/between-trial variance)

for (c in 1:(nt-1)) {

for (k in (c+1):nt) {

lhr[c,k] <- (d[k] - d[c])

log(hr[c,k]) <- lhr[c,k]

}

}

} # *** PROGRAM ENDS

**FE Rates**

# Binomial likelihood, cloglog link

# Fixed effects model

model{ # *** PROGRAM STARTS

for(i in 1:ns){ # LOOP THROUGH STUDIES

mu[i] ~ dnorm(0,.0001) # vague priors for all trial baselines

for (k in 1:na[i]) { # LOOP THROUGH ARMS

r[i,k] ~ dbin(p[i,k],n[i,k]) # Binomial likelihood

cloglog(p[i,k]) <- log(time[i]) + mu[i] + d[t[i,k]] - d[t[i,1]] # model for linear predictor

rhat[i,k] <- p[i,k] * n[i,k] # expected value of the numerators

dev[i,k] <- 2 * (r[i,k] * (log(r[i,k])-log(rhat[i,k]))

+ (n[i,k]-r[i,k]) * (log(n[i,k]-r[i,k]) - log(n[i,k]-rhat[i,k]))) #Deviance contribution

}

resdev[i] <- sum(dev[i,1:na[i]]) # summed residual deviance contribution for this trial

}

totresdev <- sum(resdev[]) #Total Residual Deviance

d[1]<-0 # treatment effect is zero for reference treatment

for (k in 2:nt){ d[k] ~ dnorm(0,.0001) } # vague priors for treatment effects

for (c in 1:(nt-1)) {

for (k in (c+1):nt) {

lhr[c,k] <- (d[k] - d[c])

log(hr[c,k]) <- lhr[c,k]

}

}

} # *** PROGRAM ENDS

**Appendix 5: Studies Excluded from NMA**

Several studies identified for this review were excluded from meta-analyses for specific reasons retailed to interventions, population and methods for endpoint evaluation. The table provided below details key findings from these studies with regard to efficacy and completion assessments in order to provide this additional information for interested readers.

| **Study** | **Rationale for Exclusion from 1 or both NMAs** | **Findings, Efficacy** | **Findings, Completion** |
| --- | --- | --- | --- |
| Park^3^ 2016 | Efficacy data NR; inconsistent criteria for completion | NR | - Totals of 12/22 (41%) in the INH-9 group and 16/21 (76%) in the RIF-4 group completed therapy. - 3 of the 12 in the INH-9 group had been tagged as “non-completers” but were still on therapy at or beyond 9 months. |
| Belknap^4^ 2015 | Comparison judged not relevant to review objectives, compared DOT vs. self-administered vs. self-administered with electronic reminders for the same treatment regimen.  Efficacy data NR; inconsistent criteria for completion | NR | - Totals of 285/328 (87%) in the 3HP DOT group, 238/320 (74%) in the self-administered 3HP group, and 236/313 (75%) in the self-administered with electronic reminders group. |
| Biraro^5^ 2015 | Efficacy data NR; inconsistent criteria for completion | NR | - Totals of 18/24 (25%) in the INH-6 group completed treatment and 17/23 (26%) in the no treatment group completed 6 months of follow-up. - Treatment completion was not specifically defined only that patients not lost to follow-up “received 6 months of INH.” Assumes that all patients who completed follow-up also took all medication. |
| Villarino^6^ 2015 | Avoidance of double-counting patients (some are also in Sterling 2011) | - Totals of 3/132 (1%) in the INH-9 group and 0/539 (0%) in the INH/RPT-3 group were confirmed or probable cases of TB. - TB confirmed by culture or diagnosed clinically based on TB diagnostic criteria of the American Thoracic Society and CDC. | - Totals of 351/434 (81%) in the INH-9 group and 415/471 (88%) in the INH/RPT-3 group completed therapy. |
| Spyridis^7^ 2007 | Unclear and potentially variable approaches in study to assess efficacy. Inconsistent criteria for completion (combination of urine test strip positivity, follow-up appointment attendance and the number of telephone reminders) | - Totals of 48/200 (24%) in the INH-9 group of 7–11-year-old children, 26/220 (12%) in the INH/RIF-4 group of 7–11-year-old children, 30/221 (14%) in the INH/RIF-4 group of 3–7-year-old children, and 23/209 (11%) in the INH/RIF-3 group of 3–7-year-old children were confirmed or probable cases of TB. - Only those with excellent to moderate compliance were assessed for efficacy after 4 months of treatment. - TB was diagnosed by chest x-ray only. | - Totals of 200/232 (86%) in the INH-9 group of 7–11-year-old children, 220/238 (92%) in the INH/RIF-4 group of 7–11-year-old children, 221/236 (94%) in the INH/RIF-4 group of 3–7-year-old children, and 209/220 (95%) in the INH/RIF-3 group of 3–7-year-old children completed therapy. - For this review, completion was defined as “excellent” or “moderate” compliance. Compliance assessed by a combination of urine test strips and appointment attendance. |
| White^8^ 2012 | Prison population with potentially misrepresentative completion data due to population movement | NR | - Totals of 47/184 (26%) in the INH-9 group and 60/180 (33%) in the RIF-4 group completed therapy. - All patients not lost to follow-up completed therapy; however, 74% and 67% lost to follow-up in each group, respectively, due to loss/withdrawal (21% of patients), deportation/transfer (49% of patients), or stopped by physician (0.5% of patients). |
| Chan^9^ 2012 | Prison population with potentially misrepresentative completion data due to population movement | NR | - Totals of 142/183 (78%) in the INH-6 group and 163/190 (86%) in the RIF-4 group completed therapy. - Patients in the RIF-4 group were more likely to complete treatment after adjusting for hepatitis B status, hepatitis C status, age ≥35 years, and prison term >2 years (p < 0.001). - Prison term >2 years was a predictor of completion. A prison term of <2 years was associated with a higher probability of parole, and participants who were paroled were 10 times more likely to drop out compared to non-paroled participants. |
| Sanchez Arcilla^10^ 2004 | Homeless population with potentially misrepresentative completion data | NR | - Totals of 22/78 (28%) in the INH-6 group and 40/65 (62%) in the RIF/PZA-2 group completed therapy. - 11/89 randomized to the INH-6 group and 19/84 randomized to the RIF/PZA-2 group did not take a single dose. - “Completion” not defined. |
| Jimenez Fuentes^11^ 2013 | Inconsistent approach to assessment of completion (involving urine strips)  Low sensitivity/specificity to diagnose TB. | - Totals of 1/294 (0.3%) in the INH-6 group and 1/294 (0.3%) in the RIF-3 group were confirmed or probable cases of TB. - Patients were telephoned 5 years after start of treatment and asked if they had developed TB. If that was not possible, public health records were assessed. | - Totals of 154/294 (52%) in the INH-6 group and 213/296 (72%) in the INH/RIF-3 group completed treatment. |
| Portilla^12^ 2003 | Comparison judged not relevant to review objectives (INH for 6-12 months, daily vs twice weekly) | - Totals of 0/16 (0%) in the INH-6- or -12-month daily group and 0/21 (0%) in the INH-6- or -12-month twice weekly group were confirmed or probable cases of TB. | - Totals of 7/16 (44%) in the INH-6- or -12-month daily group and 14/21 (67%) in the INH-6 or 12 month twice weekly group completed therapy. - Only DOT doses included in assessment of completion—all doses given DOT in twice weekly group, but not in daily group. |
| Matteelli^13^ 2000 | Comparison judged not relevant to review objectives, comparing three administration strategies of INH-6 (supervised vs unsupervised vs twice weekly) | NR | - Totals of 6/82 (7%) in the INH-6 twice weekly DOT group, 19/73 (26%) in the INH-6 twice weekly unsupervised group, and 22/53 (42%) in the INH-6 daily unsupervised group completed therapy. |
| Magdorf^14^ 1994 | Inconsistent criteria for completion (interview, urine test strips, urine color assessment, prescription frequency) | - Totals of 0/50 (0%) in the INH-6 group, 1/50 (2%) in the RIF-4 group, and 0/50 (0%) in the RIF/PZA-2 group were confirmed or probable cases of TB. - TB was diagnosed by chest x-ray only. | - Totals of 3/50 (6%) in the INH-6 group, 7/50 (14%) in the RIF-4 group, and 3/50 (6%) in the RIF/PZA-2 group completed therapy. |
| Debre^15^ 1973 | Variable duration of treatment in the INH arm (from 5 months to more than 12 months. Variable patient follow-up (3–10 years). | - Totals of 15/1519 (1%) in the INH≥5 group and 39/1451 (3%) in the untreated group were confirmed or probable cases of TB. - Cases were suspected based on clinical signs and chest x-rays. Not all suspected cases had bacteriological testing. | - Totals of 1428/1519 (94%) in the INH≥5 group completed therapy and 1427/1451 (98%) in the no treatment group completed follow-up. |

**Appendix 6: Detailed Summary of Study Characteristics**

| **Overview of Study Characteristics (in reverse chronologic order by publication date)** | | | | | | | | | | | |
| --- | --- | --- | --- | --- | --- | --- | --- | --- | --- | --- | --- |
| **Study (Year)** | **N Total** | **Regimens Compared** | **Average FU (months)*** | **Average age (yrs)** | **% female** | **Additional population features** | **Funding Source** | **Reported Efficacy?** | **Reported Completion?** | **Included in NMA of:** | |
|  |  |  |  |  |  |  |  |  |  | **Efficacy?** | **Completion?** |
| Park^3^ 2016 | 43 | INH-9;  RFMP-4 | Between  1-9 | NR | 80 | N/A | NFP | _ | √ |  |  |
| Biraro^5^ 2015 | 47 | no INH-6;  INH-6 | Unclear | NR | 63.8 | N/A | Mixed | _ | √ |  |  |
| Belknap^4^ 2015 | 1002 | INH/RFMP-3, DOT;  INH/RFMP-3, SAT  INH/RFMP-3, eSAT | Unclear | NR | 48 | N/A | NR | _ | √ |  |  |
| Martinez-Alfaro^16^ 2015 | 196 | INH-9;  INH/RFMP 3-4 | 17.5 | 37.0 | 52.6 | HIV | NR | √ | √ | √ | √ |
| Kim^17^ 2015 | 263 | No trt;  INH-9 | 21.7 | 47.9 | 34.5 | Transplant patients | NFP | √ |  | √ |  |
| Sterling^18,19^  2011, 2015 | 7,552 | INH-9;  INH/RPT-3 | 31.0 | 35.5 | 45.5 | N/A | Sanofi | √ | √ | √ | √ |
| Villarino^6^ 2015^**^ | 1,032 | INH-9;  INH/RPT-3 | 30.8 | 11 | 49.3 | Children | Sanofi | √ | √ |  |  |
| Jimenez-Fuentes^11^ 2013 | 590 | INH-6;  INH/RFMP-3 | 60.0 | 26.1 | 32.2 | N/A | SSPTS | √ | √ | √ |  |
| White^8^ 2012 | 107 | INH-9;  RFMP-4 | Unclear | NR | 7 | Prison-based | NFP |  | √ |  |  |
| Chan^9^ 2012 | 373 | INH-6;  RFMP-4 | Unclear | 30.4 | 0 | Prison-based | NFP |  | √ |  |  |
| Martinson^20^ 2011 | 1,150 | INH-6; INH-72; INH/RFMP-3;  INH/RPT-3 | 47.7 | 30.3 | 83.3 | HIV | NFP | √ | √ | √ | √ |
| Menzies^21,22^ 2008 | 847 | INH-9;  RFMP-4 | 9*** | 33.0 | 47.5 | N/A | NFP |  | √ |  | √ |
| Paloma Geijo^23^ 2007 | 96 | INH-6;  INH/RFMP-3 | 60.0 | 42.7 | 44.8 | N/A | NR | √ | √ | √ | √ |
| Spyridis^7^ 2007 | 926 | INH-9;  INH/RFMP-4; INH/RFMP-3 | Between  84-132 | 8.7 | 48.6 | Children | NFP | √ | √ |  |  |
| Schechter^24^ 2006 | 399 | INH/RPT-3;  RFMP/PZA-2 | 32.4 | 37.4 | 59.6 | N/A | NR | √ | √ | √ | √ |
| Tortajada^25^ 2005 | 352 | INH-6;  RFMP/PZA-2 | Unclear | NR | 49.0 | N/A | NR | √ | √ | √ | √ |
| Menzies^26^ 2004 | 116 | INH-9;  RFMP-4 | Unclear | 33.8 | 48.0 | N/A | NFP |  | √ |  | √ |
| Sanchez-Arcilla^10^ 2004 | 172 | INH-6;  RFMP/PZA-2 | Unclear | 42.3 | 50.5 | Homeless | NR |  | √ |  |  |
| Leung^27^ 2003 | 76 | INH-6;  RFMP/PZA-2 | 120.0 | 42.6 | 1.4 | N/A | NR |  | √ |  |  |
| Portilla^12^ 2003 | 37 | INH-6 or 12 (daily);  INH-6 or 12 (2x/week) | 24.0 | 32 | 24.3 | N/A | NFP | √ | √ |  |  |
| Johnson^28,29^ 2001, 1997 | 2,736 | PL-6; INH-6; INH/RFMP-3; INH/RFMP/PZA-3 | 22.8 | 29.5 | 68.8 | HIV | NFP | √ | √ | √ | √ |
| Matteelli^13^ 2000 | 208 | INH-6 (multiple dosing strategies tested) | 18.0 |  | 38.5 | N/A | NFP |  | √ |  |  |
| Gordin^30,31^ 2000 | 1,583 | INH-12-72;  RFMP/PZA-2 | 37.2 | 37.4 | 28.5 | HIV | Mixed | √ | √ | √ | √ |
| Martinez-Alfaro^32^ 2000 | 133 | INH-12-72;  INH/RFMP-3/4 | 17.5 | 32.2 | 24.0 | HIV | NFP | √ | √ | √ | √ |
| Halsey^33^ 1998 | 750 | INH-6;  RFMP/PZA-2 | 30.0 | 31.0 | 68.4 | HIV | NFP | √ | √ | √ | √ |
| Cowie^34^ 1996 | 382 | PL-3;  INH/RFMP/PZA-3 | 48.0 | 47.2 | 0 | Silicosis | NR | √ | √ | √ | √ |
| Magdorf^14^ 1994 | 150 | INH-6;  RFMP-4 | 24.0 | 3.6 | 58.0 | Children | NR | √ | √ |  |  |
| IUATC^35^ 1982 | 27,830 | PL-3; PL-6;  PL-12; INH-3; INH-6; INH-12 | 60.0 | 50.0 | 47.0 | N/A | NR | √ | √ | √ | √ |
| Debre^15^ 1973 | 2,970 | No trt:  INH>5 | 88.0 | NR | 45.4 | N/A | NFP | √ | √ |  |  |
| Veening^36^ 1968 | 261 | PL-12;  INH-12 | 48.0 | NR | 0 | Military | NR | √ |  | √ |  |
| Egsmose^37^ 1965 | 383 | PL-12;  INH-12 | 24.2 | NR | NR | N/A | NFP | √ |  | √ |  |
| Study sample size, primary patient demographics of interest, and study funding are noted. Checkmarks are used to identify (1) endpoints reported by each study; and (2) studies included in NMAs for the efficacy and completion endpoints.  *denotes that where a summary measure of average follow-up was not reported, one was estimated based on available data in the publication.  **denotes that this study contains a large % of pediatric patients that were also included in Sterling et al; thus considered a companion article and not included in analyses^8^  ***denotes that report assessed harms and completion, and notes patients seen each month for the first 4 months therapy and at 6 week intervals thereafter at physicians’ discretion  **Regimen-related abbreviations**: DOT= directly observed treatment; e SAT=electronic reminders + self administered treatment; INH=isoniazid; PL=placebo; PZA=pyrazinamide; RFMP=rifampin; RPT=rifapentine; SAT= self administered treatment  **Other abbreviations**: FU=follow-up; HIV=human immunodeficiency virus; IUATC=International Union Against Tuberculosis Committee; N/A=not applicable; NFP=not for profit; NMA=network meta-analysis; NR=not reported; SSPTS=Spanish Society of Pneumology and Thoracic Surgery. | | | | | | | | | | | |

**Appendix 7: Summary of Risk of Bias Assessments**

Findings of risk of bias assessment are summarized below. Regarding coloring in the table, red cells indicate high risk of bias, yellow cells indicate unclear risk of bias, green cells indicate low risk of bias, and blue cells indicate the assessment was not applicable as the indicated outcome was not evaluated.

| **Author (year)** | **Selection bias** | | **Performance bias** | | | **Reporting bias** | **Other bias** | **Efficacy-specific criteria** | **AE-specific criteria** | | | **Overall Risk of bias** | |
| --- | --- | --- | --- | --- | --- | --- | --- | --- | --- | --- | --- | --- | --- |
|  | **Random sequence generation** | **Allocation concealment** | **Blinding: patients & personnel** | **Blinding: outcome assessor** | **Attrition: Incomplete outcome data** | **Selective outcome reporting** | **Confounders balanced between groups** | **Testing regimen** | **Follow up Length** | **Follow up Frequency** | **Independent Assessment of Harms** | **Efficacy** | **Adverse events** |
| Park 2016 |  |  |  |  |  |  |  |  |  |  |  |  |  |
| Biraro 2015 |  |  |  |  |  |  |  |  |  |  |  |  |  |
| Martinez Alfaro 2015 |  |  |  |  |  |  |  |  |  |  |  |  |  |
| Kim 2015 |  |  |  |  |  |  |  |  |  |  |  |  |  |
| Sterling 2011 |  |  |  |  |  |  |  |  |  |  |  |  |  |
| Villarino 2015 |  |  |  |  |  |  |  |  |  |  |  |  |  |
| Jimenez-Fuentes 2013 |  |  |  |  |  |  |  |  |  |  |  |  |  |
| White 2012 |  |  |  |  |  |  |  |  |  |  |  |  |  |
| Chan 2012 |  |  |  |  |  |  |  |  |  |  |  |  |  |
| Martinson 2011 |  |  |  |  |  |  |  |  |  |  |  |  |  |
| Menzies 2008 |  |  |  |  |  |  |  |  |  |  |  |  |  |
| Paloma-Geijo 2007 |  |  |  |  |  |  |  |  |  |  |  |  |  |
| Spyridis 2007 |  |  |  |  |  |  |  |  |  |  |  |  |  |
| Schechter 2006 |  |  |  |  |  |  |  |  |  |  |  |  |  |
| Tortajada 2005 |  |  |  |  |  |  |  |  |  |  |  |  |  |
| Menzies 2004 |  |  |  |  |  |  |  |  |  |  |  |  |  |
| Sanchez Arcilla 2004 |  |  |  |  |  |  |  |  |  |  |  |  |  |
| Leung 2003 |  |  |  |  |  |  |  |  |  |  |  |  |  |
| Portilla 2003 |  |  |  |  |  |  |  |  |  |  |  |  |  |
| Johnson 2001 |  |  |  |  |  |  |  |  |  |  |  |  |  |
| Matteelli 2000 |  |  |  |  |  |  |  |  |  |  |  |  |  |
| Gordin 2000 |  |  |  |  |  |  |  |  |  |  |  |  |  |
| Martinez Alfaro 2000 |  |  |  |  |  |  |  |  |  |  |  |  |  |
| Halsey 1998 |  |  |  |  |  |  |  |  |  |  |  |  |  |
| Cowie 1996 |  |  |  |  |  |  |  |  |  |  |  |  |  |
| Magdorf 1994 |  |  |  |  |  |  |  |  |  |  |  |  |  |
| IUATC 1982 |  |  |  |  |  |  |  |  |  |  |  |  |  |
| Debre 1973 |  |  |  |  |  |  |  |  |  |  |  |  |  |
| Veening 1968 |  |  |  |  |  |  |  |  |  |  |  |  |  |
| Egsmose 1965 |  |  |  |  |  |  |  |  |  |  |  |  |  |

One study (Belknap, 2015) could not be formally assessed because it was published in abstract form.

**Appendix 8:**

**Numbers of Studies Per Comparison and Patients Per Treatment for Primary Analyses**

**Efficacy**

| **Comparison** | **# Studies** | **# Patients** |
| --- | --- | --- |
| **PL / no trt vs. INH-9** | **1** | **263** |
| **INH-6 vs. INH 12-72mon** | **2** | **14,375** |
| **INH-INH/RPT-3 vs. INH/RPT-3** | **1** | **655** |
| **INH-6 vs. INH/RIF-3/4** | **4** | **2,434** |
| **INH 12-72mon vs. INH/RPT-3** | **1** | **492** |
| **INH 12-INH/RIF-3/42mon vs. INH/RIF-3/4** | **2** | **626** |
| **INH/RPT-3 vs. INH/RIF-3/4** | **1** | **657** |
| **INH/RPT-3 vs. RIF/PZA-2** | **1** | **399** |
| **INH-6 vs. RIF/PZA-2** | **2** | **1,136** |
| **PL / no trt vs. INH-6** | **2** | **14,955** |
| **PL / no trt vs. INH/RIF-3/4** | **1** | **1,020** |
| **PL / no trt vs. INH/RIF/PZA-3** | **2** | **1,308** |
| **INH-6 vs. INH/RIF/PZA-3** | **1** | **998** |
| **INH/RIF-3/4 vs. INH/RIF/PZA-3** | **1** | **1,018** |
| **INH 12-72mon vs. RIF/PZA-2** | **1** | **1,583** |
| **INH-9 vs. INH/RPT-3** | **1** | **7,731** |
| **PL / no trt vs. INH 3/4** | **1** | **13,946** |
| **PL / no trt vs. INH 12-72mon** | **3** | **14,553** |
| **INH INH-6/4 vs. INH-6** | **1** | **13,921** |
| **INH 3/4 vs. INH 12-72mon** | **1** | **13,875** |
| **INH-9 vs. INH/RIF-3/4** | **1** | **196** |

| Treatment | # Studies | # Patients |
| --- | --- | --- |
| **Placebo-12** | **1** | **2302** |
| **Placebo-6** | **2** | **3,125** |
| **Placebo-3** | **2** | **2,541** |
| **INH 6 months** | **6** | **8837** |
| **INH 9 months** | **4** | **4323** |
| **INH 12-72 months** | **3** | **7775** |
| **RFPN + INH** | **3** | **4520** |
| **RIFAMPIN + PZA** | **4** | **1517** |
| **INH + RIFAMPIN + PZA** | **2** | **653** |
| **INH+RIFAMPIN 3/4 months** | **5** | **1103** |
| **RIFAMPIN** | **2** | **476** |
| **INH3-4** | **1** | **6956** |

**Completion**

| **Comparison** | **# Studies** | **# Patients** | **# Events** |
| --- | --- | --- | --- |
| **INH 6 months vs. RFPN + INH** | **1** | **655** | **588** |
| **INH 6 months vs. INH+RIFAMPIN 3/4 months** | **3** | **2,239** | **2,004** |
| **RFPN + INH vs. INH+RIFAMPIN 3/4 months** | **1** | **657** | **626** |
| **INH 9 months vs. RIFAMPIN** | **2** | **956** | **672** |
| **RFPN + INH vs. RIFAMPIN + PZA** | **1** | **399** | **373** |
| **Placebo-6 vs. INH 6 months** | **2** | **11,021** | **8,868** |
| **Placebo-6 vs. INH + RIFAMPIN + PZA** | **1** | **1,249** | **1,111** |
| **Placebo-6 vs. INH+RIFAMPIN 3/4 months** | **1** | **1,343** | **1,212** |
| **INH 6 months vs. INH + RIFAMPIN + PZA** | **1** | **1,393** | **1,237** |
| **INH + RIFAMPIN + PZA vs. INH+RIFAMPIN 3/4 months** | **1** | **1,018** | **931** |
| **INH 9 months vs. RFPN + INH** | **1** | **7,731** | **5,858** |
| **Placebo-3 vs. INH + RIFAMPIN + PZA** | **1** | **382** | **287** |
| **Placebo-1Placebo-6 vs. Placebo-6** | **1** | **4,640** | **3,505** |
| **Placebo-12 vs. Placebo-3** | **1** | **4,652** | **3,727** |
| **Placebo-12 vs. INH 6 months** | **1** | **9,267** | **7,021** |
| **Placebo-12 vs. INH 12-72 months** | **1** | **9,221** | **6,293** |
| **Placebo-INH3-4 vs. INH3-4** | **1** | **9,258** | **7,640** |
| **Placebo-6 vs. Placebo-3** | **1** | **4,688** | **4,056** |
| **Placebo-INH 12-72 months vs. INH 12-72 months** | **1** | **9,257** | **6,622** |
| **Placebo-6 vs. INH3-4** | **1** | **9,294** | **7,969** |
| **Placebo-3 vs. INH 6 months** | **1** | **9,315** | **7,572** |
| **Placebo-3 vs. INH 12-72 months** | **1** | **9,269** | **6,844** |
| **Placebo-3 vs. INH3-4** | **1** | **9,306** | **8,191** |
| **INH INH 12-72 months months vs. INH 12-72 months** | **1** | **13,884** | **10,138** |
| **INH 6 months vs. INH3-4** | **1** | **13,921** | **11,485** |
| **INH INH3-4-72 months vs. INH3-4** | **1** | **13,875** | **10,757** |
| **INH 9 months vs. INH+RIFAMPIN 3/4 months** | **1** | **196** | **166** |
| **INH 6 months vs. RIFAMPIN + PZA** | **2** | **1,102** | **738** |
| **INH 12-72 months vs. RIFAMPIN + PZA** | **1** | **1,583** | **1,180** |
| **INH 12-72 months vs. INH+RIFAMPIN 3/4 months** | **1** | **133** | **81** |

| **Treatment** | **# Studies** | **# Patients** |
| --- | --- | --- |
| **Placebo-12** | **1** | **2302** |
| **Placebo-6** | **2** | **3,125** |
| **Placebo-3** | **2** | **2,541** |
| **INH 6 months** | **6** | **8837** |
| **INH 9 months** | **4** | **4323** |
| **INH 12-72 months** | **3** | **7775** |
| **RFPN + INH** | **3** | **4520** |
| **RIFAMPIN + PZA** | **4** | **1517** |
| **INH + RIFAMPIN + PZA** | **2** | **653** |
| **INH+RIFAMPIN 3/4 months** | **5** | **1103** |
| **RIFAMPIN** | **2** | **476** |
| **INH3-4** | **1** | **6956** |

**Appendix 9: Summary of Results from Pairwise Meta-Analysis**

The tables below provide information regarding direct evidence (estimates and I^2^ values) first for the efficacy analysis, and secondly for the completion rates analysis; estimates for the most inclusive completion analysis are provided (i.e. 80-100% criteria inclusive). Results from network meta-analysis are also provided. Note that these tables do not encompass complete results for all comparisons from network meta-analysis; these are provided in the league tables shown in the main text as well as other appendices in this supplement. Results from the RE informative NMA are shown to facilitate comparisons with meta-analyses of the direct evidence.

| **Rate Ratios, Treatment Efficacy** | | | | | |
| --- | --- | --- | --- | --- | --- |
| **Comparison** | | **From Analysis of Direct Information** | | | **RR Estimates from RE Informative Model (95% CrI)** |
| **Ctrl** | **Trt** | **# studies** | **RE direct estimate,**  **RR (95% CI)** | **I2** |  |
| Pl/No trt | INH 3-4 | 1 | 0.79 (0.58-1.06) | 0 | 0.81 (0.28-2.23) |
| Pl/No trt | INH-6 | 2 | 0.51 (0.22-1.19) | 83 | 0.41 (0.19-0.80) |
| Pl/No trt | INH-9 | 1 | 0.15 (0.01-2.93) | 0 | 0.49 (0.07-1.59) |
| Pl/No trt | INH 12-72 | 3 | 0.25 (0.09-0.64) | 3 | 0.24 (0.11-0.46) |
| Pl/No trt | INH/RFMP 3-4 | 1 | 0.46 (0.27-0.77) | 0 | 0.49 (0.19-0.99) |
| Pl/No trt | INH/RFMP/PZA-3 | 2 | 0.42 (0.16-1.06) | 0 | 0.38 (0.15-0.86) |
| INH 3-4 | INH-6 | 1 | 0.45 (0.30-0.67) | 0 | 0.52 (0.18-1.40) |
| INH 3-4 | INH 12-72 | 1 | 0.32 (0.20-0.50) | 0 | 0.29 (0.10-0.79) |
| INH-6 | INH 12-72 | 2 | 0.52 (0.20-1.31) | 51 | 0.57 (0.28-1.12) |
| INH-6 | INH/RPT-3 | 1 | 1.02 (0.57-1.82) | 0 | 0.83 (0.29-2.02) |
| INH-6 | INH/RFMP 3-4 | 4 | 1.06 (0.46-2.44) | 72 | 1.18 (0.52-2.33) |
| INH-6 | RFMP/PZA-2 | 1 | 1.32 (0.66-2.64) | 0 | 0.75 (0.28-1.75) |
| INH-6 | INH/RFMP/PZA-3 | 1 | 0.61 (0.33-1.11) | 0 | 0.92 (0.35-2.37) |
| INH-9 | INH/RPT-3 | 1 | 0.47 (0.21-1.04) | 0 | 0.71 (0.25-3.08) |
| INH-9 | INH/RFMP 3-4 | 1 | 2.53 (0.1-62.02) | 0 | 1.01 (0.29-5.56) |
| INH 12-72 | INH/RPT-3 | 1 | 2.92 (1.31-6.50) | 0 | 1.45 (0.51-3.70) |
| INH 12-72 | INH/RFMP 3-4 | 2 | 2.83 (0.90-8.91) | 84 | 2.04 (0.89-4.55) |
| INH 12-72 | RFMP/PZA-2 | 1 | 1.00 (0.59-1.68) | 0 | 1.30 (0.51-3.13) |
| INH/RPT-3 | INH/RFMP 3-4 | 1 | 2.17 (1.23-3.83) | 0 | 1.43 (0.53-4.00) |
| INH/RPT-3 | RFMP/PZA-2 | 1 | 0.36 (0.04-3.46) | 0 | 0.91 (0.29-2.87) |
| INH/RFMP 3-4 | INH/RFMP/PZA-3 | 1 | 0.98 (0.51-1.89) | 0 | 0.78 (0.30-2.29) |

Comparison of estimates from traditional pairwise meta-analysis and RE informative network meta-analysis for pairs of interventions where direct data was available. Estimates expressed are rate ratios with 95% CI/CrI.

NA=not applicable (only 1 study available); INH=isoniazid; PL=placebo; RPT=rifapentine; RIF=rifampin; PZA=pyrazinamide; trt=treatment; RCT=randomized controlled trial; RR=rate ratio; RE=random effects; FE=fixed effects; CI=confidence interval; CrI=credible interval.

| **Odds Ratios, Treatment Completion** | | | | | |
| --- | --- | --- | --- | --- | --- |
| **Comparison** | | **From Analysis of Direct Information** | | | **OR Estimates from RE Informative Model (95% CrI)** |
| **Ctrl** | **Trt** | **# studies** | **RE direct estimate,**  **OR (95% CI)** | **I2** |  |
| INH/RFMP/PZA-3 | INH/RFMP 3-4 | 1 | 1.46 (0.94-2.27) | 0 | 1.33 (0.66-2.64) |
| INH/RPT-3 | INH/RFMP 3-4 | 1 | 0.82 (0.40-1.69) | 0 | 0.88 (0.45-1.72) |
| INH/RPT-3 | RFMP/PZA-2 | 1 | 1.10 (0.50-2.44) | 0 | 0.68 (0.34-1.39) |
| INH-12 | INH/RFMP 3-4 | 1 | 1.28 (0.64-2.58) | 0 | 2.69 (1.44-4.71) |
| INH-12 | RFMP/PZA-2 | 1 | 1.87 (1.48-2.36) | 0 | 2.09 (1.16-3.56) |
| INH 3-4 | INH-12 | 1 | 0.47 (0.42-0.52) | 0 | 0.39 (0.20-0.81) |
| INH 3-4 | INH-6 | 1 | 0.53 (0.48-0.58) | 0 | 0.50 (0.25, 0.96) |
| INH-6 | INH/RFMP/PZA-3 | 1 | 1.17 (0.82-1.68) | 0 | 1.59 (0.85-3.04) |
| INH-6 | INH/RFMP 3-4 | 3 | 2.43 (1.46-4.04) | 57 | 2.11 (1.31-3.46) |
| INH-6 | INH/RPT-3 | 1 | 4.34 (2.36-7.99) | 0 | 2.41 (1.26-4.91) |
| INH-6 | INH-12 | 1 | 0.60 (0.56-0.65) | 0 | 0.78 (0.48-1.39) |
| INH-6 | RFMP/PZA-2 | 2 | 1.55 (0.92-2.61) | 91 | 1.64 (1.04-2.70) |
| INH-9 | INH/RFMP 3-4 | 1 | 4.00 (1.08-14.83) | 0 | 1.92 (0.90-4.23) |
| INH-9 | INH/RPT-3 | 1 | 2.06 (1.85-2.29) | 0 | 2.19 (1.14-4.30) |
| INH-9 | RFMP 3-4 | 2 | 2.38 (1.29-4.37) | 0 | 2.41 (1.26-4.91) |
| Placebo-12 | INH-12 | 1 | 0.96 (0.86-1.06) | 0 | 1.16 (0.59-2.45) |
| Placebo-12 | INH 3-4 | 1 | 3.01 (2.69-3.37) | 0 | 3.01 (1.39-6.42) |
| Placebo-12 | INH-6 | 1 | 1.59 (1.44-1.77) | 0 | 1.49 (0.73-2.89) |
| Placebo-12 | Placebo-3 | 1 | 4.56 (3.86-5.39) | 0 | 4.17 (1.96-8.60) |
| Placebo-12 | Placebo-6 | 1 | 2.05 (1.78-2.35) | 0 | 1.94 (0.95-3.88) |
| Placebo-3 | INH/RFMP/PZA-3 | 1 | 0.73 (0.46-1.17) | 0 | 0.57 (0.30-1.10) |
| Placebo-3 | INH-12 | 1 | 0.21 (0.18-0.24) | 0 | 0.28 (0.15-0.57) |
| Placebo-3 | INH 3-4 | 1 | 0.66 (0.56-0.77) | 0 | 0.72 (0.35-1.52) |
| Placebo-3 | INH-6 | 1 | 0.35 (0.30-0.41) | 0 | 0.36 (0.19-0.66) |
| Placebo-6 | INH/RFMP/PZA-3 | 1 | 1.15 (0.80-1.68) | 0 | 1.22 (0.62-2.40) |
| Placebo-6 | INH/RFMP 3-4 | 1 | 1.69 (1.14-2.49) | 0 | 1.63(0.88-2.99) |
| Placebo-6 | INH-12 | 1 | 0.47 (0.42-0.52) | 0 | 0.60 (0.34-1.15) |
| Placebo-6 | INH 3-4 | 1 | 1.47 (1.3-1.67) | 0 | 1.55 (0.78-3.15) |
| Placebo-6 | INH-6 | 2 | 0.87 (0.54-1.39) | 52 | 0.77 (0.45-1.30) |
| Placebo-6 | Placebo-3 | 1 | 2.23 (1.87-2.66) | 0 | 2.16 (1.13-4.10) |

Comparison of estimates from traditional pairwise meta-analysis and RE informative network meta-analysis for pairs of interventions where direct data was available.

NA=not applicable (only 1 study available); INH=isoniazid; PL3=placebo 3 months; PL6=placebo 6 months; PL12=placebo 12 months; RPT=rifapentine; RIF=rifampin; PZA=pyrazinamide; RCT=randomized controlled trial; OR=odds ratio; RE=random effects; FE=fixed effects; CI=confidence interval; CrI=credible interval.

**Primary Data**

**Efficacy**

| **General Study Information** | | | | | **Group 1 data** | | | **Group 2 data** | | | **Group 3 data** | | | **Group 4 data** | | |
| --- | --- | --- | --- | --- | --- | --- | --- | --- | --- | --- | --- | --- | --- | --- | --- | --- |
| **Study** | **Group 1** | **Group 2** | **Group 3** | **Group 4** | **n1** | **p1** | **f1** | **n2** | **p2** | **f2** | **n3** | **p3** | **f3** | **n4** | **p4** | **f4** |
| Kim 2015 | PL/No trt | INH-9 | NA | NA | 132 | 2.27 | 245.3 | 131 | 0 | 231.4 | NA | NA | NA | NA | NA | NA |
| Martinson 2011 | INH-6 | INH 12-72 | INH/RPT-3 | INH/RMP 3-4 | 327 | 6.73 | 1143.9 | 164 | 4.88 | 1187.5 | 328 | 7.32 | 1219.7 | 329 | 7.29 | 561 |
| Schechter 2006 | INH/RPT-3 | RFMP/PZA-2 | NA | NA | 206 | 1.46 | 564 | 193 | 0.52 | 522 | NA | NA | NA | NA | NA | NA |
| Tortajada 2005 | INH-6 | RFMP/PZA-2 | NA | NA | 199 | 0 | 99.5 | 153 | 0 | 25.5 | NA | NA | NA | NA | NA | NA |
| Johnson 2001 | PL/No trt | INH-6 | INH/RFMP 3-4 | INH/RFMP/PZA-3 | 464 | 9.05 | 1012 | 536 | 6.34 | 1101 | 556 | 3.96 | 1151 | 462 | 3.25 | 800 |
| Gordin 2004 | INH 12-72 | RFMP/PZA-2 | NA | NA | 792 | 3.66 | 2416.7 | 791 | 3.54 | 2333.3 | NA | NA | NA | NA | NA | NA |
| Sterling 2011 | INH-9 | INH/RPT-3 | NA | NA | 3745 | 0.48 | 9619 | 3986 | 0.23 | 10327 | NA | NA | NA | NA | NA | NA |
| Cowie 1996 | PL/No trt | INH/RFMP/PZA-3 | NA | NA | 191 | 7.85 | 3040.1 | 191 | 5.76 | 5889.2 | NA | NA | NA | NA | NA | NA |
| Halsey 1998 | INH-6 | RFMP/PZA-2 | NA | NA | 392 | 3.57 | 925 | 392 | 4.85 | 950 | NA | NA | NA | NA | NA | NA |
| IUATC 1982 | PL/No trt | INH 3-4 | INH-6 | INH 12-72 | 6990 | 1.39 | 33971.4 | 6956 | 1.09 | 33806.2 | 6965 | 0.49 | 33849.9 | 6919 | 0.35 | 33626.3 |
| Jimenez 2013 | INH-6 | INH/RFMP 3-4 | NA | NA | 294 | 0.34 | 990 | 296 | 0.34 | 915 | NA | NA | NA | NA | NA | NA |
| Alfaro 1998 | INH-9 | INH/RFMP 3-4 | NA | NA | 98 | 0 | 130.7 | 98 | 1.02 | 155.2 | NA | NA | NA | NA | NA | NA |
| Veening 1968 | PL/No trt | INH 12-72 | NA | NA | 128 | 9.38 | 745.9 | 133 | 0.75 | 775.1 | NA | NA | NA | NA | NA | NA |
| Paloma Geijo 2007 | INH-6 | INH/RFMP 3-4 | NA | NA | 45 | 2.22 | 225 | 51 | 0 | 255 | NA | NA | NA | NA | NA | NA |
| Egsmose 1965 | PL/No trt | INH 12-72 | NA | NA | 188 | 1.6 | 360.3 | 195 | 1.03 | 409.1 | NA | NA | NA | NA | NA | NA |
| Alfaro 2016 | INH 12-72 | INH/RFMP 3-4 | NA | NA | 64 | 6.25 | 94.6 | 69 | 2.9 | 96.2 | NA | NA | NA | NA | NA | NA |

**Treatment Completion**


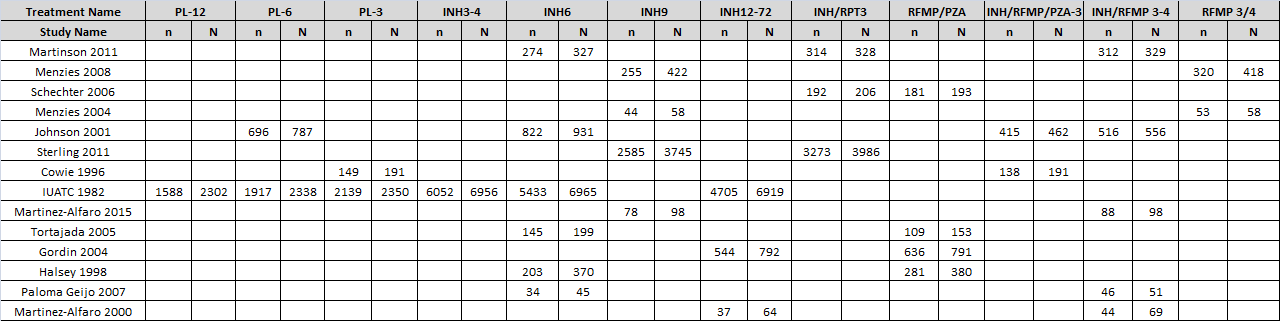


**Appendix 10: Results From Sensitivity Analyses**

| **Sensitivity Analyses, Efficacy/Prevention of Active TB (16 RCTs; 44,149 patients)** | | | | | | |
| --- | --- | --- | --- | --- | --- | --- |
| **Intervention (versus placebo/no trt)** | **Model** | **RR (95% CrI),**  **Primary**  **Analysis** | **Meta-Regression Analysis Adjusting for:** | | | |
|  |  |  | **Mean Age**  **(years)** | **Year of Publication** | **Study Population is HIV-infected** | **Population had Silicosis or Transplant** |
| INH 3-4 | RE inf  RE vague  FE | 0.81 (0.28-2.23)  0.80 (0.18-3.29)  0.82 (0.61-1.09) | 0.97 (0.29-3.00)  0.96 (0.18-4.94)  1.01 (0.67-1.52) | 0.83 (0.17-2.80)  0.78 (0.09-4.53)  0.99 (0.60-1.66) | 0.75 (0.22-2.44)  0.72 (0.12-3.73)  0.78 (0.58-1.04) | 0.83 (0.25-2.71)  0.82 (0.16-4.32)  0.82 (0.61-1.10) |
| INH-6 | RE inf  RE vague  FE | 0.41 (0.19-0.80)  0.40 (0.14-1.00)  0.42 (0.32-0.55) | 0.45 (0.20-0.92)  0.43 (0.14-1.22)  0.48 (0.34-0.66) | 0.42 (0.14-0.96)  0.39 (0.09-1.25)  0.48 (0.32-0.73) | 0.36 (0.14-0.91)  0.33 (0.08-1.10)  0.37 (0.26-0.51) | 0.43 (0.18-0.98)  0.42 (0.13-1.32)  0.43 (0.32-0.56) |
| INH-9 | RE inf  RE vague  FE | 0.49 (0.07-1.59)  0.36 (0.03-1.67)  0.62 (0.26-1.46) | 0.48 (0.07-1.79)  0.38 (0.02-2.02)  0.68 (0.28-1.67) | 0.43 (0.05-1.64)  0.32 (0.02-1.71)  0.67 (0.27-1.66) | 0.40 (0.05-1.59)  0.28 (0.01-1.56)  0.54 (0.23-1.34) | 0.48 (0.06-2.15)  0.38 (0.02-2.55)  0.64 (0.29-1.61) |
| INH 12-72 | RE inf  RE vague  FE | 0.24 (0.11-0.46)  0.22 (0.08-0.54)  0.24 (0.17-0.34) | 0.25 (0.11-0.51)  0.23 (0.08-0.62)  0.28 (0.18-0.42) | 0.23 (0.06-0.61)  0.21 (0.03-0.82)  0.29 (0.17-0.47) | 0.21 (0.08-048)  0.20 (0.05-0.58)  0.22 (0.15-0.31) | 0.24 (0.10-0.52)  0.23 (0.07-0.66)  0.25 (0.17-0.34) |
| INH/RPT-3 | RE inf  RE vague  FE | 0.35 (0.10-0.88)  0.31 (0.07-1.11)  0.37 (0.21-0.61) | 0.36 (0.10-1.00)  0.33 (0.06-1.30)  0.41 (0.23-0.71) | 0.34 (0.07-0.98)  0.29 (0.04-1.28)  0.41 (0.23-0.74) | 0.30 (0.08-0.94)  0.26 (0.04-1.15)  0.32 (0.18-0.56) | 0.36 (0.09-1.14)  0.33 (0.06-1.53)  0.37 (0.22-0.63) |
| INH/RFMP 3-4 | RE inf  RE vague  FE | 0.49 (0.19-0.99)  0.45 (0.13-1.18)  0.52 (0.36-0.75) | 0.51 (0.19-1.09)  0.46 (0.13-1.34)  0.55 (0.37-0.79) | 0.48 (0.14-1.13)  0.42 (0.08-1.40)  0.57 (0.38-0.86) | 0.39 (0.13-1.11)  0.36 (0.07-1.28)  0.42 (0.26-0.67) | 0.51 (0.18-1.20)  0.47 (0.12-1.52)  0.54 (0.37-0.77) |
| RFMP/PZA-2 | RE inf  RE vague  FE | 0.31 (0.10-0.79)  0.29 (0.06-1.01)  0.32 (0.19-0.52) | 0.32 (0.10-0.89)  0.31 (0.06-1.20)  0.36 (0.21-0.61) | 0.31 (0.06-0.96)  0.27 (0.03-1.30)  0.37 (0.20-0.67) | 0.27 (0.07-0.85)  0.24 (0.04-1.07)  0.28 (0.17-0.47) | 0.31 (0.09-0.95)  0.30 (0.06-1.34)  0.32 (0.19-0.52) |
| INH/RFMP/PZA-3 | RE inf  RE vague  FE | 0.38 (0.15-0.86)  0.36 (0.11-1.12)  0.39 (0.24-0.61) | 0.41 (0.16-1.02)  0.40 (0.11-1.46)  0.41 (0.26-0.65) | 0.37 (0.13-0.92)  0.35 (0.09-1.27)  0.41 (0.25-0.64) | 0.32 (0.11-0.97)  0.30 (0.06-1.26)  0.32 (0.19-0.53) | 0.46 (0.14-1.69)  0.47 (0.09-2.88)  0.43 (0.24-0.72) |
| **Model DIC; resdev;**  **# data points** | RE inf  RE vague  FE | **203.9; 41.9; 38**  **200.9; 38.0; 38**  **213.2; 57.3; 38** | **203.6; 41.2; 38**  **200.8; 37.5; 38**  **213.1; 56.2; 38** | **204.1; 41.2; 38**  **201.8; 38.04; 38**  **214.1; 57.3; 38** | **203.2; 40.6; 38**  **200.9; 37.4; 38**  **213.5; 56.1; 38** | **203.5; 40.6; 38**  **200.9; 37.4; 38**  **215.0; 58.05; 38** |
| **Beta value** | RE inf  RE vague  FE | **NA**  **NA**  **NA** | **-0.02 (-0.08 to 0.03)**  **-0.03 (-0.10 to 0.04)**  **-0.02 (-0.04 to 0.01)** | **0.001 (-0.06 to 0.05)**  **-0.002 (-0.07 to 0.06)**  **0.01 (-0.01 to 0.04)** | **0.40 (-1.40 to 1.69)**  **0.51 (-1.39 to 2.71)**  **0.39 (-0.13 to 0.91)** | **-0.48 (-2.52 to 1.16)**  **-0.59 (-3.43 to 1.54)**  **-0.29 (-1.21 to 0.63)** |
| **SD** | RE inf  RE vague | **0.44 (0.02-1.06)**  **0.66 (0.25-1.49)** | **0.47 (0.02-1.15)**  **0.72 (0.28-1.63)** | **0.50 (0.02-1.20)**  **0.74 (0.29-1.71)** | **0.51 (0.04-1.21)**  **0.75 (0.28-1.82)** | **0.51 (0.04-1.24)**  **0.75 (0.30-1.77)** |

Summary rate ratios (RR) versus placebo/no treatment are shown from the RE informative, RE vague and FE analyses for both the primary efficacy analysis and subsequent sensitivity analyses performed using meta-regression. Model fit measures, regression coefficients and the between study variance parameter are also reported.

**Sensitivity Analysis: RE vague and FE NMAs, Efficacy (# of active TB cases)**


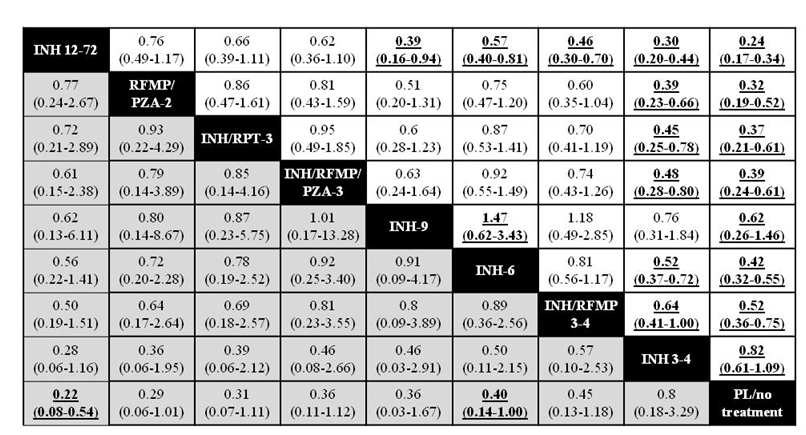


A complete summary of estimates from sensitivity analyses using RE vague and FE network meta-analyses for treatment efficacy is provided where efficacy was analyzed as a rate endpoint. Statistically significant differences between regimens are shown in bold, underlined font. Treatments are ordered from upper left to lower right in order of decreasing SUCRA value from the random effects analysis, which is shown in the lower triangle. Results from the RE informative analysis were provided in the main text.

To draw interpretations from both models, the lower/right-most comparison for each comparison is the reference treatment.

Model fit results for the RE vague model were resdev=38.0 (versus 38 data points), DIC=200.9; for the FE model, corresponding values were resdev=57.3 (versus 38 data points), DIC=213.2.

***Abbreviations***. INH=isoniazid; RPT=rifapentine; RFMP=rifampin; PZA=pyrazinamide; PL=placebo.

**Sensitivity Analysis: RE informative, RE vague and FE NMAs, Efficacy (# of active TB cases) As a Binary Endpoint**

**A: RE Informative Analysis B: RE vague (lower) and FE (upper) Analyses**

| 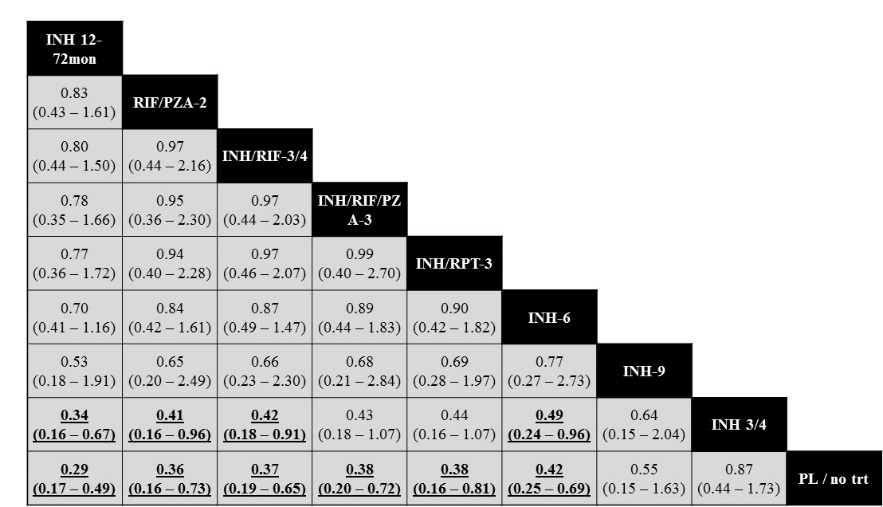 | 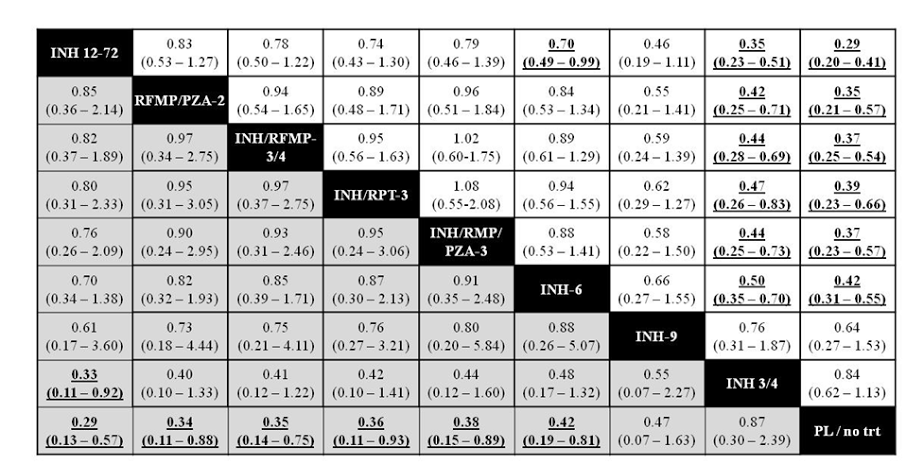 |
| --- | --- |

A complete summary of estimates from sensitivity analyses using RE informative (figure A), RE vague and FE network meta-analyses (figure B) for treatment efficacy is provided where efficacy was treated as a binary endpoint (as opposed to a rate). Statistically significant differences between regimens are shown in bold, underlined font. Treatments are ordered from upper left to lower right in order of decreasing SUCRA value from the RE informative (figure A) and RE vague (figure B) analyses, which are shown in the lower triangles of the two league tables.

To draw interpretations from both models, the lower/right-most comparison for each comparison is the reference treatment.

Model fit results for the RE informative model were resdev=36.33 (compared to 35 data points), DIC=274.53. For the RE vague model, corresponding values were resdev=35.22, DIC=274.06. For the FE model, the corresponding valules were resdev=63.93, DIC=295.29.

***Abbreviations***. INH=isoniazid; RPT=rifapentine; RFMP=rifampin; PZA=pyrazinamide.

**Sensitivity Analysis: RE vague and FE NMAs, Treatment Completion**


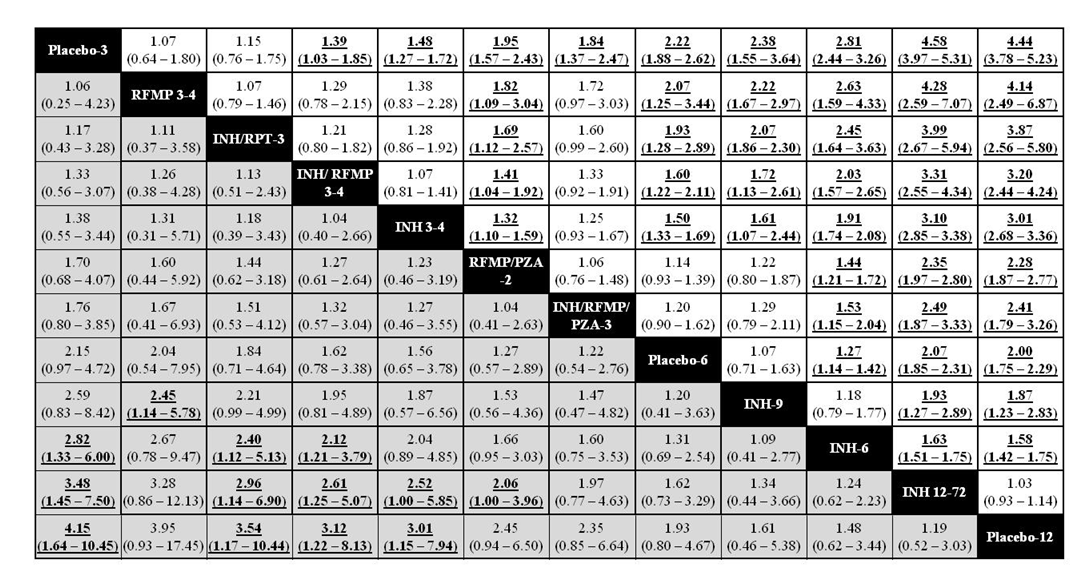


A complete summary of estimates from sensitivity analyses using RE vague and FE network meta-analyses for treatment completion is provided where completion was analyzed as a binary endpoint. Results from the RE informative model were presented in the main text. Statistically significant differences between regimens are shown in bold, underlined font. Treatments are ordered from upper left to lower right in order of decreasing SUCRA value from the random effects analysis, which is shown in the lower triangle.

To draw interpretations from both models, the lower/right-most comparison for each comparison is the reference treatment.

Model fit results for the RE vague model were resdev=35.22 (compared to 35 data points), DIC=274.06. For the FE model, the corresponding values were resdev=63.93, DIC=295.29.

***Abbreviations***. INH=isoniazid; RPT=rifapentine; RFMP=rifampin; PZA=pyrazinamide; PL=placebo.

| **Network Diagrams, Additional Sensitivity Analyses for Treatment Completion** | |
| --- | --- |
| **Limited to studies with 80%-90% completion criteria** | **80-100% criteria, with additional studies from Stagg et al where patients without a confirmed LTBI diagnosis were also included** |
| 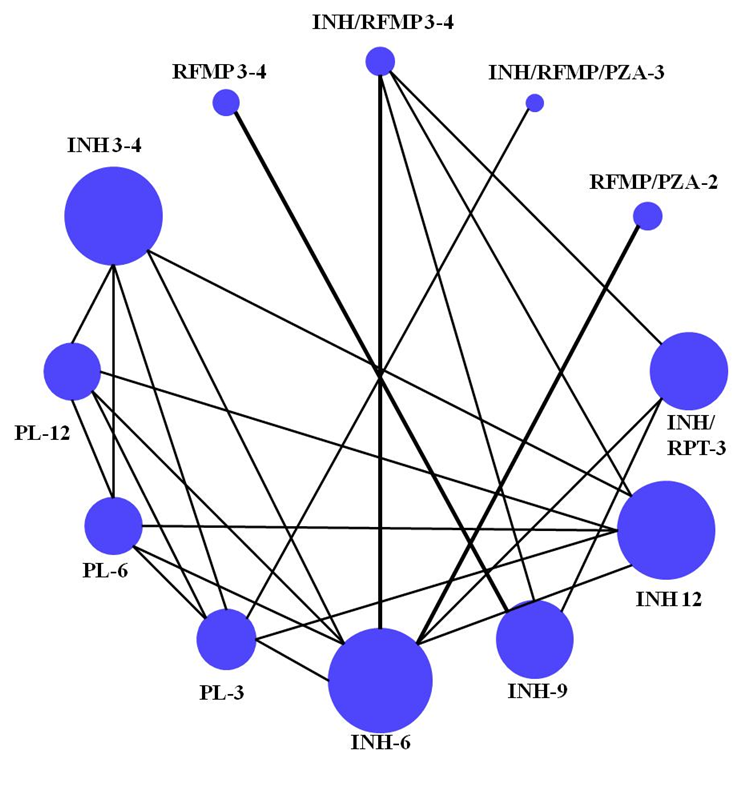  **11 studies, 39,410 patients** | 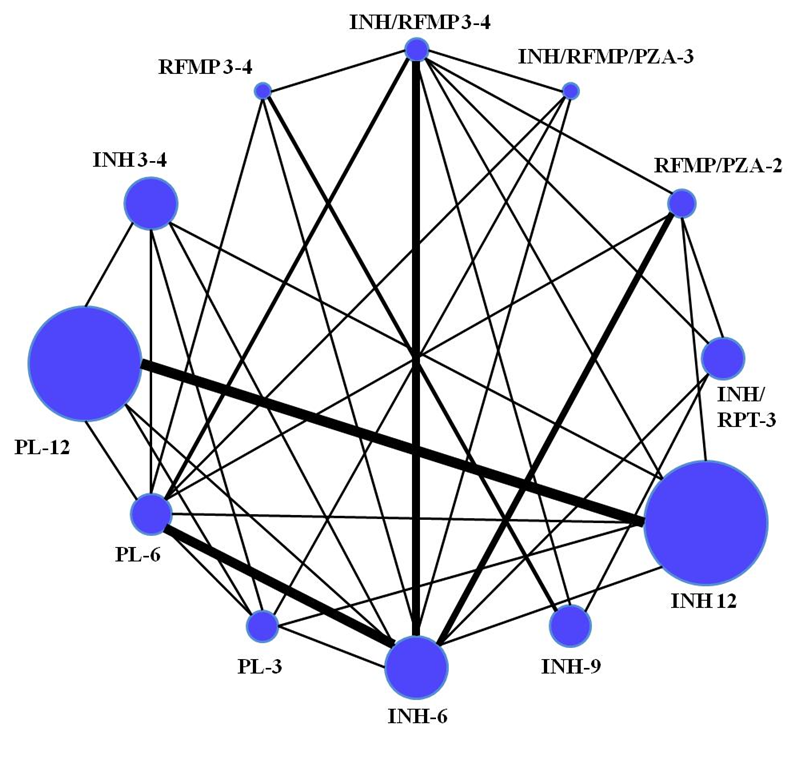  **25 studies, 108,947 patients** |

| **Findings from Additional Sensitivity Analyses, Completion Rate NMA** | | | |  |
| --- | --- | --- | --- | --- |
| **Intervention** | **Model** | **Within-Study Completion Rate Definitions between:** | | **80-100% + studies from past review without a confirmed LTBI diagnosis** |
|  |  | **80-100%** | **80-90%** |  |
| ***# of included studies (patients)*** | N/A | 14 (44,128) | 11 (39,410) | 25 (108, 947) |
| Placebo-3 | RE informative  RE vague  FE | 4.17 (1.96, 8.60);  4.15 (1.64, 10.50);  4.44 (3.78, 5.23) | 4.58 (1.44, 14.68);  4.59 (0.80, 27.80);  4.56 (3.87, 5.39) | 4.25 (2.67, 6.49);  3.66 (2.20, 5.87);  3.96 (3.43, 4.58) |
| INH 3-4 | RE informative  RE vague  FE | 3.01 (1.39, 6.42);  3.01 (1.15, 7.94);  3.01 (2.68, 3.36) | 3.02 (0.95, 9.66);  3.02 (0.53, 18.40);  3.01 (2.69, 3.37) | 3.05 (1.91, 4.75);  2.64 (1.57, 4.34);  2.68 (2.46, 2.93) |
| INH/RPT-3 | RE informative  RE vague  FE | 3.58 (1.40, 8.83);  3.54 (1.17, 10.40);  3.87 (2.56, 5.80) | 3.82 (0.80, 16.56);  3.61 (0.38, 32.9);  4.88 (2.78, 8.71) | 3.07 (1.71, 5.36)  2.62 (1.38, 4.80);  2.87 (1.98, 4.18) |
| RFMP/PZA-2 | RE informative  RE vague  FE | 2.44 (1.11, 5.36);  2.45 (0.94, 6.50);  2.28 (1.87, 2.77) | 2.00 (0.56, 7.56);  1.88 (0.22, 15.10);  2.82 (2.14, 3.74) | 2.12 (1.46, 3.00:  1.83 (1.19, 2.74);  1.88 (1.62, 2.18) |
| INH/RFMP/PZA-3 | RE informative  RE vague  FE | 2.36 (1.02, 5.40);  2.35 (0.85, 6.64);  2.41 (1.79, 3.26) | 3.34 (0.62, 18.42);  3.36 (0.27, 45.60);  3.35 (2.02, 5.48) | 2.34 (1.37, 3.90);  2.01 (1.12, 3.52);  2.12 (1.59, 2.84) |
| INH/RFMP 3-4 | RE informative  RE vague  FE | 3.14 (1.43, 6.77);  3.12 (1.22, 8.13);  3.20 (2.44, 4.24) | 3.25 (0.90, 11.60);  3.22 (0.49, 22.3);  3.66 (2.43, 5.54) | 2.64 (1.74, 3.92);  2.27 (1.42, 3.54);  2.45 (1.95, 3.08) |
| RFMP 3-4 | RE informative  RE vague  FE | 3.95 (1.15, 13.72);  3.95 (0.93, 17.50);  4.14 (2.49, 6.87) | 3.76 (0.52, 23.02);  3.49 (0.21, 49.60);  5.23 (2.76, 10.00) | 2.81 (1.37, 5.63);  2.35 (1.11, 4.97);  2.92 (1.86, 4.59) |
| Placebo-6 | RE informative  RE vague  FE | 1.94 (0.95, 3.88);  1.93 (0.80, 4.67);  2.00 (1.75, 2.29) | 2.06 (0.65, 6.51);  2.06 (0.36, 12.60);  2.05 (1.79, 2.35) | 1.95 (1.35, 2.72);  1.68 (1.11, 2.46);  1.78 (1.61, 1.97) |
| INH-6 | RE informative  RE vague  FE | 1.49 (0.73, 2.89);  1.48 (0.62, 3.44);  1.58 (1.42, 1.75) | 1.31 (0.41, 3.79);  1.25 (0.23, 6.73);  1.58 (1.43, 1.76) | 1.57 (1.11, 2.15);  1.35 (0.91, 1.95);  1.41 (1.32, 1.52) |
| INH-9 | RE informative  RE vague  FE | 1.64 (0.57, 4.45);  1.61 (0.46, 5.38);  1.87 (1.23, 2.83) | 1.51 (0.25, 7.05);  1.37 (0.11, 13.30);  2.35 (1.33, 4.24) | 1.32 (0.68, 2.42);  1.11 (0.55, 2.14);  1.38 (0.95, 2.02) |
| INH-12 | RE informative  RE vague  FE | 1.16 (0.59, 2.45);  1.19 (0.52, 3.03);  0.97 (0.87, 1.07) | 1.18 (0.41, 3.84);   1.23 (0.24, 7.21);  0.96 (0.87, 1.06) | 0.87 (0.71, 1.08);  0.87 (0.70, 1.09);  0.83 (0.81, 0.86) |
| DIC (RE inf; RE vague; FE)  Resdev (RE inf; RE vague; FE)  # data points | N/A  N/A  N/A | 274.54; 274.1; 295.3  36.3; 35.2; 63.9  35 | 211.9; 211.8; 228.6  27.4; 26.7; 47.7  27 | 491.9; 490.8; 579.1  65.7; 64.0; 168.2  61 |

Summary ORs versus placebo/no treatment for the regimens of primary interest are shown from the RE informative, RE vague and FE analyses for both the primary analysis and subsequent sensitivity analyses.

**Appendix 11: Model Fit Results from Primary Network Meta-Analyses**

The adequacy of statistical fit for a network meta-analysis model can be established by comparing the number of data points in the data set (i.e. the total number of arms across studies) to the posterior residual deviance (‘resdev’) for the corresponding analysis; approximately a 1-1 ratio should be present, meaning they should be of similar value. To compare the fit of competing models, their deviance information criteria (‘DIC’) values can be compared, with smaller values being preferable and differences of 4-5 points or more reflecting an important difference. We have reported fixed and random effects (with both vague and informative prior distributions on the between study variance parameter as described in the report’s main text) consistency model results in this report, and below a summary of model fit information has been provided. Model fit results for sensitivity analyses are presented in the preceding appendices.

| **Summary of Model Fit Measures from Primary Network Meta-Analyses** | | | | |
| --- | --- | --- | --- | --- |
| **Model** | **# data points** | **Resdev** | **Sd** | **DIC** |
| **Efficacy (Active TB)** | | | | |
| Re informative consistency | 38 | 41.9 | 0.44 (0.02-1.06) | 203.9 |
| RE vague consistency | 38 | 38.0 | 0.66 (0.25-1.49) | 200.9 |
| FE consistency | 38 | 57.4 | NA | 213.2 |
| RE informative inconsistency | 38 | 37.9 | 0.10 (0.001-0.78) | 201.2 |
| RE vague inconsistency | 38 | 36.3 | 0.52 (0.08-2.07) | 199.9 |
| FE inconsistency | 38 | 49.3 | NA | 209.9 |
| **Regimen Completion (80-100% rates)** | | | | |
| Re informative consistency | 35 | 36.3 | 0.33 (0.16-0.63) | 274.5 |
| RE vague consistency | 35 | 35.2 | 0.41 (0.20-0.83) | 274.1 |
| FE consistency | 35 | 63.9 | NA | 295.3 |
| RE informative inconsistency | 35 | 34.9 | 0.64 (0.16-2.86) | 275.8 |
| RE vague inconsistency | 35 | 35.1 | 0.65 (0.16-2.98) | 275.8 |
| FE inconsistency | 35 | 43.3 | NA | 281.7 |

NA=not applicable (Sd is not an estimable parameter in fixed effects analyses)

**Appendix 12: PRISMA NMA Checklist**

***Source****:* *The PRISMA extension statement for reporting of systematic reviews incorporating network meta-analyses of health care interventions: checklist and explanations.*Hutton B, Salanti G, Caldwell DM, Chaimani A, Schmid CH, Cameron C, Ioannidis JP, Straus S, Thorlund K, Jansen JP, Mulrow C, Catalá-López F, Gøtzsche PC, Dickersin K, Boutron I, Altman DG, Moher D.Ann Intern Med. 2015 Jun 2;162(11):777-84.

| **Section/Topic** | **Item #** | **Checklist Item** | **Reported on Page #** |
| --- | --- | --- | --- |
| **TITLE** |  |  |  |
| Title | 1 | Identify the report as a systematic review *incorporating a network meta-analysis (or related form of meta-analysis).* | 1 |
|  |  |  |  |
| **ABSTRACT** |  |  |  |
| Structured summary | 2 | Provide a structured summary including, as applicable:  **Background:** main objectives  **Methods:** data sources; study eligibility criteria, participants, and interventions; study appraisal; and *synthesis methods, such as network meta-analysis.*  **Results:** number of studies and participants identified; summary estimates with corresponding confidence/credible intervals; *treatment rankings may also be discussed. Authors may choose to summarize pairwise comparisons against a chosen treatment included in their analyses for brevity.*  **Discussion/Conclusions:** limitations; conclusions and implications of findings.  **Other:** primary source of funding; systematic review registration number with registry name. | 2 |
|  |  |  |  |
| **INTRODUCTION** |  |  |  |
| Rationale | 3 | Describe the rationale for the review in the context of what is already known*, including mention of why a network meta-analysis has been conducted.* | 4 |
| Objectives | 4 | Provide an explicit statement of questions being addressed, with reference to participants, interventions, comparisons, outcomes, and study design (PICOS). | 4 |
|  |  |  |  |
| **METHODS** |  |  |  |
| Protocol and registration | 5 | Indicate whether a review protocol exists and if and where it can be accessed (e.g., Web address); and, if available, provide registration information, including registration number. | 5 |
| Eligibility criteria | 6 | Specify study characteristics (e.g., PICOS, length of follow-up) and report characteristics (e.g., years considered, language, publication status) used as criteria for eligibility, giving rationale. *Clearly describe eligible treatments included in the treatment network, and note whether any have been clustered or merged into the same node (with justification).* | 5-6 |
| Information sources | 7 | Describe all information sources (e.g., databases with dates of coverage, contact with study authors to identify additional studies) in the search and date last searched. | 5 |
| Search | 8 | Present full electronic search strategy for at least one database, including any limits used, such that it could be repeated. | e-Appendix 1 |
| Study selection | 9 | State the process for selecting studies (i.e., screening, eligibility, included in systematic review, and, if applicable, included in the meta-analysis). | 5 |
| Data collection process | 10 | Describe method of data extraction from reports (e.g., piloted forms, independently, in duplicate) and any processes for obtaining and confirming data from investigators. | 6 |
| Data items | 11 | List and define all variables for which data were sought (e.g., PICOS, funding sources) and any assumptions and simplifications made. | 6 |
| **Geometry of the network** | **S1** | Describe methods used to explore the geometry of the treatment network under study and potential biases related to it. This should include how the evidence base has been graphically summarized for presentation, and what characteristics were compiled and used to describe the evidence base to readers. | 6 |
| Risk of bias within individual studies | 12 | Describe methods used for assessing risk of bias of individual studies (including specification of whether this was done at the study or outcome level), and how this information is to be used in any data synthesis. | 6 |
| Summary measures | 13 | State the principal summary measures (e.g., risk ratio, difference in means). *Also describe the use of additional summary measures assessed, such as treatment rankings and surface under the cumulative ranking curve (SUCRA) values, as well as modified approaches used to present summary findings from meta-analyses.* | 7 |
| Planned methods of analysis | 14 | Describe the methods of handling data and combining results of studies for each network meta-analysis. This should include, but not be limited to:   - *Handling of multi-arm trials;* - *Selection of variance structure;* - *Selection of prior distributions in Bayesian analyses; and* - *Assessment of model fit.* | 6-7 |
| **Assessment of Inconsistency** | **S2** | Describe the statistical methods used to evaluate the agreement of direct and indirect evidence in the treatment network(s) studied. Describe efforts taken to address its presence when found. | e-appendix 3 |
| Risk of bias across studies | 15 | Specify any assessment of risk of bias that may affect the cumulative evidence (e.g., publication bias, selective reporting within studies). | e-appendix 3 |
| Additional analyses | 16 | Describe methods of additional analyses if done, indicating which were pre-specified. This may include, but not be limited to, the following:   - Sensitivity or subgroup analyses; - Meta-regression analyses; - *Alternative formulations of the treatment network; and* - *Use of alternative prior distributions for Bayesian analyses (if applicable).* | 8 |
|  |  |  |  |
| **RESULTS†** |  |  |  |
| Study selection | 17 | Give numbers of studies screened, assessed for eligibility, and included in the review, with reasons for exclusions at each stage, ideally with a flow diagram. | 8-9; e-appendix 2 |
| **Presentation of network structure** | **S3** | Provide a network graph of the included studies to enable visualization of the geometry of the treatment network. | Figures 1a, 1b |
| **Summary of network geometry** | **S4** | Provide a brief overview of characteristics of the treatment network. This may include commentary on the abundance of trials and randomized patients for the different interventions and pairwise comparisons in the network, gaps of evidence in the treatment network, and potential biases reflected by the network structure. | 10 |
| Study characteristics | 18 | For each study, present characteristics for which data were extracted (e.g., study size, PICOS, follow-up period) and provide the citations. | 9-10; e-appendix 6 |
| Risk of bias within studies | 19 | Present data on risk of bias of each study and, if available, any outcome level assessment. | 9; e-appendix 7 |
| Results of individual studies | 20 | For all outcomes considered (benefits or harms), present, for each study: 1) simple summary data for each intervention group, and 2) effect estimates and confidence intervals. *Modified approaches may be needed to deal with information from larger networks.* | e-appendix 9 |
| Synthesis of results | 21 | Present results of each meta-analysis done, including confidence/credible intervals. *In larger networks, authors may focus on comparisons versus a particular comparator (e.g. placebo or standard care), with full findings presented in an appendix. League tables and forest plots may be considered to summarize pairwise comparisons.* If additional summary measures were explored (such as treatment rankings), these should also be presented. | 11-13; additional information in e-appendices 7-9 |
| **Exploration for inconsistency** | **S5** | Describe results from investigations of inconsistency. This may include such information as measures of model fit to compare consistency and inconsistency models, *P* values from statistical tests, or summary of inconsistency estimates from different parts of the treatment network. | e-appendix 9 |
| Risk of bias across studies | 22 | Present results of any assessment of risk of bias across studies for the evidence base being studied. | NA |
| Results of additional analyses | 23 | Give results of additional analyses, if done (e.g., sensitivity or subgroup analyses, meta-regression analyses*, alternative network geometries studied, alternative choice of prior distributions for Bayesian analyses,* and so forth). | 13;  e-appendices 10-11 |
|  |  |  |  |
| **DISCUSSION** |  |  |  |
| Summary of evidence | 24 | Summarize the main findings, including the strength of evidence for each main outcome; consider their relevance to key groups (e.g., healthcare providers, users, and policy-makers). | 13-14 |
| Limitations | 25 | Discuss limitations at study and outcome level (e.g., risk of bias), and at review level (e.g., incomplete retrieval of identified research, reporting bias). *Comment on the validity of the assumptions, such as transitivity and consistency. Comment on any concerns regarding network geometry (e.g., avoidance of certain comparisons).* | 16 |
| Conclusions | 26 | Provide a general interpretation of the results in the context of other evidence, and implications for future research. | 17 |
|  |  |  |  |
| **FUNDING** |  |  |  |
| Funding | 27 | Describe sources of funding for the systematic review and other support (e.g., supply of data); role of funders for the systematic review. This should also include information regarding whether funding has been received from manufacturers of treatments in the network and/or whether some of the authors are content experts with professional conflicts of interest that could affect use of treatments in the network. | 17 |

PICOS = population, intervention, comparators, outcomes, study design.

* Text in italics indicates wording specific to reporting of network meta-analyses that has been added to guidance from the PRISMA statement.

† Authors may wish to plan for use of appendices to present all relevant information in full detail for items in this section.

**Appendix 13: Reference List for Appendices**

1. Stagg H *et al.* Treatment of latent tuberculosis infection: a network meta-analysis. *Ann. Intern. Med.* **161,** 419–428 (2014).

2. Brown S *et al.* A Microsoft-Excel-based tool for running and critically appraising network meta-analyses--an overview and application of NetMetaXL. *Syst. Rev.* **3:110. doi: 10.1186/2046-4053-3-110.,** (2014).

3. Park S *et al.* A prospective cohort study of latent tuberculosis in adult close contacts of active pulmonary tuberculosispatients in Korea. *Korean J Intern Med* **31,** 517–524

4. Belknap R *et al.* Adherence to Once-Weekly Self-Administered INH and Rifapentine for Latent TB: iAdhere. *Conf. Retroviruses Opportunistic Infect. 2015* **abstract 827 LB,**

5. Biraro, I. A. *et al.* Effect of isoniazid preventive therapy on immune responses to mycobacterium tuberculosis: an open label randomised, controlled, exploratory study. *BMC Infect. Dis.* **15,** 438 (2015).

6. Villarino M, Scott N, Weiss S & et al. Treatment for Preventing Tuberculosis in Children and Adolescents: A Randomized Clinical Trial of a 3-Month, 12-Dose Regimen of a Combination of Rifapentine and Isoniazid. *JAMA Pediatr.* **Epub 2015/01/13,** (2015).

7. Spyridis N *et al.* The Effectiveness of a 9-Month Regimen of Isoniazid Alone versus 3- and 4-Month Regimens of Isoniazid plus Rifampin for Treatment of Latent Tuberculosis Infection in Children: Results of an 11-Year Randomized Study. *CID* **45,** 715–722 (2007).

8. White M *et al.* Isoniazid vs. Rifampin for Latent Tuberculosis Infection in Jail Inmates: Toxicity and Adherence. *J Correct Health Care* **18,** 131–142 (2012).

9. Chan P *et al.* Latent tuberculosis infection treatment for prison inmates: a randomised controlled trial. *Int J Tuberc Lung Dis* **16,** 633–638 (2012).

10. Sanchez-Arcilla I, Vilchez JM, Garcia de la Torre M, Fernandez X & Noguerado A. Treatment of latent tuberculosis among homeless population. Comparison between two therapeutic approaches. *Med Clin Barc* **122,** 57–59 (2004).

11. Jimenez-Fuentes M, de Souza Galvao M, Auge C, Solsana Peiro J & Altet-Gomez M. Rifampicin plus isoniazid for the prevention of tuberculosis in an immigrant population. *Int J Tuberc Lung Dis* **17,** 326–332 (2013).

12. Portilla J *et al.* Directly observed treatment of latent tuberculosis infection: comparative study of two isoniazid regimens. *Enferm Infec Microbiol Clin* **21,** 293–295 (2003).

13. Matteelli J *et al.* Tolerability of twice-weekly rifabutin-isoniazid combinations versus daily isoniazid for latent tuberculosis in HIV-infected subjects: a pilot study. *Int J Tuberc Lung Dis* **3,** 1043–1046 (1999).

14. Magdorf K, Arizzi-Rusche F, Geiter L, O’Brien R & Wahn U. Compliance and tolerance of new antituberculotic short-term chemo-prevention regimes in childhood, a pilot study. *Pneumologie* **48,** 761–764 (1994).

15. Debre R, Perdrizet S, Lotte A, Naveau M & Lert F. Isoniazid Chemoprophylaxis of Latent Primary Tuberculosis: in Five Trial Centres in France from 1959 to 1969. *Int. J. Epidemiol.* **2,** 153–160 (1973).

16. Martinez Alfaro E *et al.* Compliance, tolerance and efficacy of a short course of chemoprophylaxis for tuberculosis. *Med Clin Barc* **111,** 401–404 (2015).

17. Kim SH *et al.* Isoniazid treatment to prevent TB in kidney and pancreas transplant recipients based on an interferon-g-releasing assay: an exploratory randomized controlled trial. *J Antimicrob Chemother* **70,** 1567–1572 (2015).

18. Sterling T, Villarino M, Borisov A & et al. Three months of rifapentine and isoniazid for latent tuberculosis infection. *NEJM* **365,** 2155–2166 (2011).

19. Sterling T, Moro R, Borisov A & et al. Flu-like and Other Systemic Drug Reactions Among Persons Receiving Weekly Rifapentine plus Isoniazid or Daily Isoniazid for Treatment of Latent Tuberculosis Infection in the PREVENT TB study. Clinical infectious diseases : an official publication of the Infectious Diseases Society of America. *Epub 20150424* (2015).

20. Martinson N, Barnes G, Moulton L & et al. New regimens to prevent tuberculosis in adults with HIV infection. *NEJM* **365,** 11–20 (2011).

21. Menzies D *et al.* Adverse Events with 4 Months of Rifampin Therapy or 9 Months of Isoniazid Therapy for Latent Tuberculosis Infection: A Randomized Trial. *Ann. Intern. Med.* **149,** 689–697 (2008).

22. Trajman, A. *et al.* Factors associated with treatment adherence in a randomised trial of latent tuberculosis infection treatment. *Int. J. Tuberc. Lung Dis. Off. J. Int. Union Tuberc. Lung Dis.* **14,** 551–559 (2010).

23. Paloma Geijo M *et al.* Short course isoniazid and rifampin compared with isoniazid for latent tuberculosis infection: a randomized clinical trial. *Enferm Infecc Microbiol Clin* **25,** 300–304 (2006).

24. Schechter M, Zajdenverg R, Falco G & et al. Weekly rifapentine/isoniazid or daily rifampin/pyrazinamide for latent tuberculosis in household contacts. *Am J Respir Crit Care Med* **173,** 922–926 (2006).

25. Tortajada C *et al.* Is the combination of pyrazinamide plus rifampicin safe for treating latent tuberculosis infection in persons not infected by the human immunodeficiency virus? *Int J Tuberc Lung Dis* **9,** 276–281 (2005).

26. Menzies D *et al.* Treatment Completion and Costs of a Randomized Trial of Rifampin for 4 Months versus Isoniazid for 9 Months. *Am J Respir Crit Care Med* **170,** 445–449 (2004).

27. Leung C *et al.* Initial Experience on Rifampin and Pyrazinamide vs Isoniazid in the Treatment of Latent Tuberculosis Infection Among Patients With Silicosis in Hong Kong. *Chest* **124,** 2112–2118 (2003).

28. Johnson J *et al.* Duration of efficacy of treatment of latent tuberculosis infection in HIV-infected adults. *AIDS* **15,** 2137–2147 (2001).

29. Whalen C *et al.* A trial of three regimens to prevent tuberculosis in ugandan adulsts infected with the human immunodeficiency virus. *NEJM* **337,** 801–808 (1997).

30. Gordin F, Cohn D, Matts J, Chaisson R & O’Brien R. Hepatotoxicity of Rifampin and Pyrazinamide in the Treatment of Latent Tuberculosis Infection in HIV-Infected Persons: Is It Different Than in HIV-Uninfected Persons? *Clin Infec Dis* **39,** 561–565 (2004).

31. Gordin, F. *et al.* Rifampin and pyrazinamide vs isoniazid for prevention of tuberculosis in HIV-infected persons: an international randomized trial. Terry Beirn Community Programs for Clinical Research on AIDS, the Adult AIDS Clinical Trials Group, the Pan American Health Organization, and the Centers for Disease Control and Prevention Study Group. *JAMA* **283,** 1445–1450 (2000).

32. Martinez-Alfaro E *et al.* Assessment of two chemopropyhylaxis regimens for tuberculosis in HIV-infected patients. *Med Clin Barc* **115,** 161–165 (2000).

33. Halsey N *et al.* Randomised trial of isoniazid versus rifampicin and pyrazinamide for prevention of tuberculosis in HIV-1 infection. *Lancet* **351,** 786–792 (1998).

34. Cowie R. Short course chemoprophylaxis with rifampicin, isoniazid and pyrazinamide for tuberculosis evaluated in gold miners with chronic silicosis: a double-blind placebo controlled trial. *Tuberc Lung Dis.* **77,** 239–243 (1996).

35. INTERNATIONAL UNION AGAINST TUBERCULOSIS COMMITTEE ON PROPHYLAXIS. Efficacy of various durations of isoniazid preventive therapy for tuberculosis: five years of follow-up in the IUAT trial. *Bull. World Health Organ.* **60,** 555–564 (1982).

36. Veening G. Long term isoniazid prophylaxis: controlled trial on INH prophylaxis after recent tuberculin conversion in young adults. *Bull Int Union Tuberc* **41,** 169–171 (1968).

37. Egsmose T, Ang-Awa J & Poti S. The Use of Isoniazid among Household Contacts of Open Cases of Pulmonary Tuberculosis. *Bull. World Health Organ.* **33,** 419–433 (1965).
